# A Systematic Review of Healthcare Applications for Smartphones

**DOI:** 10.1186/1472-6947-12-67

**Published:** 2012-07-10

**Authors:** Abu Saleh Mohammad Mosa, Illhoi Yoo, Lincoln Sheets

**Affiliations:** 1 University of Missouri Informatics Institute (MUII), 241 Engineering Building West, Columbia, MO, 65211, USA; 2 Health Management and Informatics (HMI) Department, University of Missouri School of Medicine, CS&E Bldg. DC006.00, Columbia, MO, 65212, USA; 3 Department of Family and Community Medicine, University of Missouri School of Medicine, M226 Medical Sciences Building, DC032.00, Columbia, MO, 65212, USA

## Abstract

**Background:**

Advanced mobile communications and portable computation are now combined in handheld devices called “smartphones”, which are also capable of running third-party software. The number of smartphone users is growing rapidly, including among healthcare professionals. The purpose of this study was to classify smartphone-based healthcare technologies as discussed in academic literature according to their functionalities, and summarize articles in each category.

**Methods:**

In April 2011, MEDLINE was searched to identify articles that discussed the design, development, evaluation, or use of smartphone-based software for healthcare professionals, medical or nursing students, or patients. A total of 55 articles discussing 83 applications were selected for this study from 2,894 articles initially obtained from the MEDLINE searches.

**Results:**

A total of 83 applications were documented: 57 applications for healthcare professionals focusing on disease diagnosis (21), drug reference (6), medical calculators (8), literature search (6), clinical communication (3), Hospital Information System (HIS) client applications (4), medical training (2) and general healthcare applications (7); 11 applications for medical or nursing students focusing on medical education; and 15 applications for patients focusing on disease management with chronic illness (6), ENT-related (4), fall-related (3), and two other conditions (2). The disease diagnosis, drug reference, and medical calculator applications were reported as most useful by healthcare professionals and medical or nursing students.

**Conclusions:**

Many medical applications for smartphones have been developed and widely used by health professionals and patients. The use of smartphones is getting more attention in healthcare day by day. Medical applications make smartphones useful tools in the practice of evidence-based medicine at the point of care, in addition to their use in mobile clinical communication. Also, smartphones can play a very important role in patient education, disease self-management, and remote monitoring of patients.

## Background

Recent years have seen an increased adoption of smartphones by healthcare professionals as well as the general public [1-6]. The smartphone is a new technology that combines mobile communication and computation in a handheld-sized device, facilitating mobile computing at the point of care. The main objective of this study is to classify the smartphone-based healthcare technologies in the literature according to their functionalities and summarize them in each category. We present a systematic literature review in this regard. To the best of our knowledge, this study is the first study for classifying and summarizing healthcare applications for smartphones in a systematic literature review format.

The healthcare system is highly mobile in nature, involving multiple clinical locations such as clinics, inpatient wards, outpatient services, emergency departments, operating theaters, intensive care units (ICUs), laboratories, etc. [7-10]. As such, working in the healthcare system requires extensive mobility of healthcare professionals as well as communication and collaboration among different individuals, including their colleagues and patients. Healthcare professionals mainly used pagers for mobile communication until the wide availability of cell phones in 1990s [11]. The advent of mobile Personal Digital Assistants (PDAs) during 1990s enabled healthcare professionals to organize their contacts and calendars electronically, adding another device in their pockets. The combined functionality of a pager, a cell phone and a PDA is now replaced by a single device called a “smartphone”, which is becoming very popular among healthcare professionals as well as the general public [12]. Further details on smartphones and their operating-system platforms are discussed in Appendix I. Table 1 in Appendix I illustrates an overview of OS features of smartphone platforms and Table 2 in Appendix I illustrates the support of common features by smartphone OS platforms with the availability of hardware in the device.

A systematic review summarizing 23 surveys on PDA usage by healthcare professionals (conducted in the U.S. (16 surveys), Canada (4 surveys), Australia (1 survey), both the U.S. and Puerto Rico (1 survey), and both the U.S. and Canada (1 survey) between 2000 and 2005) demonstrated that overall adoption rate varied between 45% and 85% in 2004–2005 [1]. The patterns of PDA usage reported by this study [1] were: (1) younger physicians (94%) were more likely than older physicians (84.5%) to use a PDA, and students and medical residents tended to be younger and were more likely to use a PDA; (2) no significant gender difference in PDA users was reported among physicians, internists or residents; (3) the biggest adopters of PDAs were family and general practitioners; (4) large-practice and hospital-based physicians were higher adopters of PDAs than office-based physicians; and (5) PDA use was more likely among urban physicians than rural physicians. According to research conducted by Manhattan Research in 2009 on the professional use of smartphones by physicians, about 64% of the physicians in the U.S. used smartphones in 2009 compared to only 30% in 2001. This report found a noticeable increase in smartphone adoption and predicted that 81% of physicians would use smartphone in the U.S. by 2012 [2].

During recent years, healthcare professionals have required access to many technologies at the point of care, such as: (1) Hospital Information Systems (HISs) including Electronic Health Record (EHR) or Electronic Medical Record (EMR) systems, Clinical Decision Support Systems (CDSSs), Picture Archiving and Communication Systems (PACSs), Laboratory Information Systems (LISs), etc.; (2) evidence-based resources such as PubMed and Up-to-Date; (3) clinical applications such as medical calculators, drug databases, and disease diagnosis applications; and (4) clinical communication such as voice calling, video conferencing, text messaging, and email messaging. Access to information systems or clinical applications in healthcare settings is mainly provided through stationary computers, which does not fully support the mobile nature of healthcare. In response, additional portable and wireless mobile information communication technologies (MICTs) such as Computers on Wheels (COWs) or Workstations on Wheels (WOWs) are used in some healthcare setup to further facilitate access to information technologies at the point of care [13]. The increased adoption of smartphones by healthcare professionals demonstrates the opportunity for improved clinical communication, and access to information systems and clinical tools at the point of care, or from anywhere at anytime [11,13-26]. Accordingly, many software applications have been produced for healthcare professionals in order to facilitate the practice of evidence-based medicine (EBM) at the point of care.

The main stakeholders in the healthcare process are healthcare consumers (patients). Consumer-oriented care, where patients are directly involved in the process of care, will greatly improve the healthcare process. Technology can play key roles in consumer-oriented healthcare (for example, making information accessible to consumers, integrating consumers’ preferences into HISs, remote monitoring, communication, etc.), which is studied in a branch of medical informatics called Consumer Health Informatics (CHI) [27]. The management of diseases with chronic conditions is very costly. For example, the report published in the 2011 National Diabetes Fact Sheet by the Centers for Disease Control and Prevention (CDCP) of the U.S. Department of Health and Human Services demonstrated that about 25.8 million people in the U.S. (8.3% of population) have diabetes and the estimated national cost (direct and indirect) of diabetes was 174 million dollars in 2007 [28]. Self-management and remote monitoring of patients are becoming viable solutions for management of diseases with chronic conditions, and smartphones are playing very important role [29-35]. Clinician-led patient education in disease prevention and management through smartphones and text-messaging is convenient and effective [36,37]. A study performed by comScore demonstrated an increase of 27.7 million smartphone users (from 49.1 million at the end of May 2010 to 76.8 million at the end of May 2011) in the U.S. [4,5]. We discuss 6 major operating systems (OS) for smartphones and their current market share and worldwide market share forecasts for 2015, and compare their features in Table 1 and Table 2 in Appendix I.

## Methods

### Data Sources

In this study, we present a systematic literature review of healthcare applications for smartphones following the PRISMA statement for systematic reviews [38]. MEDLINE citations were searched in April 2011, using the PubMed search engine, for articles that discuss the design, development, evaluation, or use of smartphone software applications to be used by healthcare professionals or patients. The MeSH vocabulary, which is used to index MEDLINE articles, does not contain “smartphone”. However, since smartphones are the successors of PDAs and handheld computers, the MeSH term "computers, handheld" was used in MEDLINE to index the articles with a focus on smartphones. In this study, the search terms used for eligible articles were: (1) "computers, handheld"[MeSH Terms], or ("computer$"[All Fields] and "handheld"[All Fields]), or "handheld computer$"[All Fields]; or (2) "smartphone$"[All Fields], or "smart-phone$"[All Fields], or "smart phone$"[All Fields], or "iPhone"[All Fields], or "Android"[All Fields], or "blackberry"[All Fields], or "black berry"[All Fields], or "Windows Mobile"[All Fields] or "Windows Phone"[All Fields]. The search terms only focused on the terms synonymously to the term “smartphone.” The search criteria did not include any limitation on publication date, and the earliest eligible article was published in 2003. The reference lists of included articles were also searched systematically.

### Inclusion and Exclusion Criteria

This study mainly focused on the functionality of software for smartphones within the scope of healthcare. As such, the inclusion criteria for the articles were the design, development, evaluation or use of smartphone-based applications for healthcare. As the search terms only focused on the terms synonymously related to the term “smartphone” and included the MeSH term "computers, handheld"[MeSH Terms], the search result included many articles that have no focus on application programs of smartphones but used smartphones for other purposes, or used handheld computer devices than smartphones or their predecessors, PDAs. Those articles were excluded from this study. This study also excluded articles that were not published in English. In short, we adopted a recall-focused retrieval strategy not to miss relevant documents in the MEDLINE. Irrelevant documents by the strategy are excluded manually.

### Study Selection and Data Extraction

The titles and abstracts of the identified citations were read to screen the articles based on the selection criteria described in the previous section. The remaining articles were read in full text to extract information from each article. The extracted information is presented in tables (Tables 3, 4, 5, 6, 7, 8, 9, 10, 11 and 12) including smartphone application names, supported operating system platforms, descriptions and functionalities of the applications. In addition, we accessed the websites of the applications to get the latest release information (last access in June 2011).

## Results

**Figure 1:**
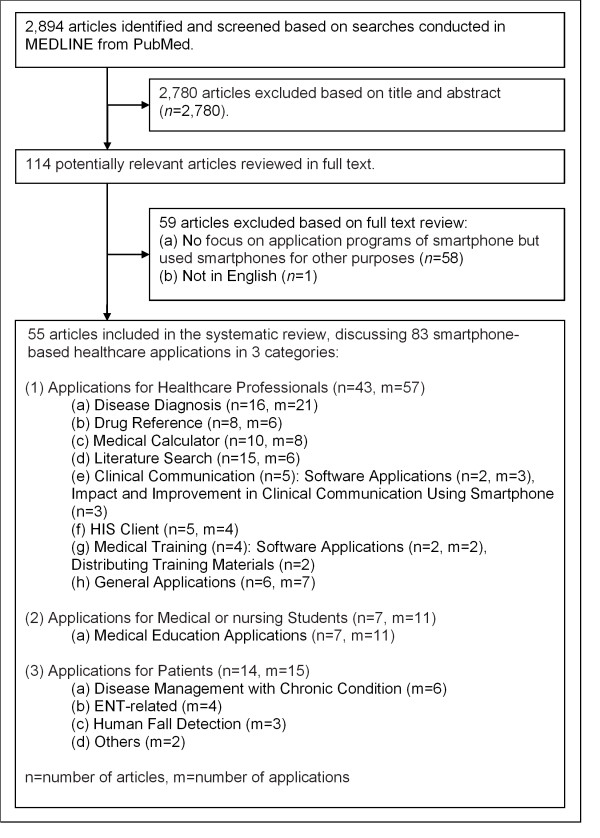
**Trial Flow Diagram.** This figure presents the trial flow diagram of identifying eligible articles for this study. A total of 2,894 articles were returned from the literature searches. Initially, a total of 2,780 articles were screened based on their titles and abstracts satisfying the inclusion and exclusion criteria. An additional 59 articles were excluded after full text review of 114 articles. Finally, 55 articles discussing 83 smartphone-based healthcare applications met the eligibility criteria. The earliest eligible articles were published in 2003, and 24 of the 55 articles were published in 2010 through April 2011.

The flow diagram of identifying eligible articles for this study is shown in Figure 1. The literature searches resulted in a total of 2,894 articles, which were then initially screened based on the titles and abstracts. The inclusion and exclusion criteria, described in the previous section, were followed in the screening process resulting in the exclusion of 2,780 articles. The remaining 114 articles were then reviewed in full text, and an additional 59 articles were excluded because they did not primarily discuss smartphone applications but used smartphones for other purposes. One article was excluded because it was not written in English. The resulting 55 articles, discussing 83 smartphone-based healthcare applications, met the eligibility criteria. The earliest eligible articles were published in 2003, and 24 of the 55 articles were published in 2010 through April 2011.

The applications were grouped by the targeted user of the applications, as divided into three groups: (1) healthcare professionals, (2) medical or nursing students, and (3) patients.

### Application for Healthcare Professionals

There are many smartphone-based applications for healthcare professionals. In this study, a total of 57 applications for healthcare professionals were identified from 43 eligible articles. These applications were grouped into 7 categories based on functional similarity: disease diagnosis, drug reference, medical calculators, literature search, clinical communication, HIS clients, and medical training. Some applications did not fall into any of those categories and were discussed in the section titled “general healthcare applications”.

#### Disease Diagnosis Applications

Disease diagnosis applications were designed to access diagnosis and treatment information in a few taps on a smartphone. A total of 21 disease diagnosis applications were discussed in 16 articles. Table 3 in Appendix II provides detailed information about the 21 applications. Of these, eight articles mainly focused on the overview and uses of applications [11,17,23,26,39-42], four articles published surveys on the use of the applications [16,43-45], two articles compared the applications in terms of treatment recommendations [46,47], and two articles investigated the application of smartphones to diagnosis and treatment [48,49].

Handheld versions of printed medical references for disease diagnosis were available on smartphones, providing information on infectious diseases, pathogens, diagnosis, treatment, medications, differential diagnosis etc. There are eight of these applications including Johns Hopkins Antibiotic Guide (JHABx), 5-Minute Clinical Consult (5MCC), 5-Minute Infectious Diseases Consult (5MIDC), Sanford Guide to Antimicrobial Therapy (SG), ePocrates ID, Infectious Disease Notes (ID Notes), Pocket Medicine Infectious Diseases (PMID), and IDdx. These applications also provide internal links for easy navigation and searching.

A 2004 study evaluated SG, JHABx, 5MIDC, 5MCC, PMID, and ePocrates ID for treatment recommendations on 202 cases and reported that five of the applications provided treatment recommendations in more than 95% cases. Specifically, SG and ePocrates ID provided treatment recommendations in every case, JHABx in 99% of cases, 5MCC in 97%, 5MID in 95%, and PMID in 52% [46]. The treatment recommendations of four applications, ePocrates ID, JHABx, 2002 SG, and ID Notes, were compared with current practice guidelines by Miller et al. (2003) [47], who concluded that JHABx is highly preferable for its inclusion of more details, accurate treatment and diagnosis information, and an automatic update process. A survey study reported that nurse practitioners ranked medical text and reference books as the second most useful category of PDA applications, and 14% of responders specifically mentioned 5MCC as very useful [43].

UpToDate is an evidence-based clinical smartphone tool that provides the most recent clinical evidence and includes more than 9,000 physicians’ topics, 5,000 drug topics, practice change updates, etc. This application is very useful in the practice of EBM at the bedside [40] and is very useful for the integration of test results with clinical information [17]. In a community hospital, internal medicine residents reported UpToDate as their most commonly used evidence-based resource and about 50% of them reported using it for general medical and scientific information as well as specific questions on patient management [45]. Phua & Lim (2008) [16] conducted a survey on the use of evidence-based resources by residents in a tertiary care hospital in Singapore with an institutional subscription to UpToDate. They found that only 69.4% of the residents were aware of their institutional subscription, so the actual use of UpToDate was low (56.7%). However, most of the users (93.4%) found it useful and would recommend UpToDate to their colleagues, and for about three-fifth of them using UpToDate led to changes in patient-management decisions [16]. One of our authors (LS), a practicing physician, has observed that his colleagues in U.S. private and academic practice consult UpToDate far more often than any other single resource at the point of care, and that it seldom fails to return fast, focused, and practical results.

There were six applications providing common laboratory test information, including reference values and interpretation, causes for abnormal (increased or decreased) values, and laboratory unit conversions. These were Palm LabDX, Normal Lab Values, Lab Unit Converter, Labs 360^o^, Davis’s Laboratory and Diagnostic Tests, and Pocket Guide to Diagnostic Tests [17,26,44]. Palm LabDX was evaluated by third-year medical students in their internal medicine clerkships, mostly during patient care activities, and found to be useful [44]. Lippi & Plebani (2011) [26] emphasized the formal review process of the laboratory applications in strict compliance with laboratory medicine professional bodies such as the International Federation of Clinical Chemistry and Laboratory Medicine (IFCC).

Smartphones can also be applied in the process of diagnosis and treatment using software application. A simple smartphone application for eye-care professionals is a visual acuity test. For example, EyeChart is an iPhone application that includes the Snellen eye chart to measure visual acuity [41]. A similar application is EyePhone, which includes a distance E-test, near visual acuity test, color test, Amsler grid, and pupil gauge test [42]. Our physician author, who frequently practices in low-resource clinical settings, has found that having convenient access to a Snellen chart is indispensable although its use is limited to patients that are literate in the Roman alphabet. The DizzyFIX application guides clinicians in the Epley Maneuver, a series of precise head and body positions that is the primary treatment of Benign Paroxysmal Positional Vertigo [41]. Mezzana et al. (2011) [49] used the Video Laser Level application in oculoplastic surgery by superimposing two digital horizontal lines onto a real image to show canthal ligament misalignment. Joundi et al. (2011) [48] measured tremor frequency using the iPhone iSeismometer application and found that it matched the more sophisticated and expensive EMG analysis.

Smartphone-based disease diagnosis applications are useful evidence-based resources at the bedside. These applications can also help clinicians in identifying appropriate laboratory tests based on symptoms, decreasing unnecessary tests and reducing cost of care. Radiology tests, for example, are very expensive and unnecessary tests are undesirable; but clinicians can use the “eRoentgen Radiology Dx” application on their smartphones to identify the most appropriate radiology exams for patients, reducing the cost of care and improving patient safety [23]. Other applications use clinical algorithms to help clinicians understand and apply principles of disease diagnosis. The flowcharts included in 5MCC and Pocket Guide to Diagnostic Tests, for example, can help physicians see at a glance diagnostic possibilities that they may have overlooked.

#### Drug Reference Applications

A total of six drug reference applications were discussed in the eight articles. Table 4 in Appendix II provides detailed information about the drug reference applications. Six of these articles mainly focused on the overview and use of the applications [11,39,40,50-52], and two articles published surveys on use of the applications [43,53]. The six applications are: Skyscape’s RxDrugs, Epocrates, Medscape, SafeMed Pocket, FDA drugs, and DrugDoses.net. The drug reference applications generally include the names of drugs, their indications, dosages, pharmacology, drug-drug interactions, contraindications, cost, and identifying characteristics. Epocrates was cited as the most commonly accessed drug-reference application [11,50,51]. Epocrates and Skyscape’s RxDrugs, another drug reference that our physician author has seen widely used in the U.S., can check multiple drug-drug interaction at the same time [11]. FDA Drugs, which includes package inserts or official labels of FDA-approved prescription and over-the-counter drugs, shares this functionality and also searches by active ingredients [39]. SafeMed Pocket, which was designed to be used in Sweden and lists drugs that are licensed for sale in that country, is integrated with a clinical decision support system (CDSS) for use by geriatiric homecare nurses to warn of drug-drug interactions, therapeutic duplications, and dosages that are unsuitable for elderly people [52].

Smartphone-based drug reference applications can be useful evidence-based resources at the point of care, as Richardson & Burdette (2003) [40] show in their example of using Epocarates in the practice of evidence-based medicine during hospital rounds. A survey of PDA usage by nurse practitioners reported that drug reference applications were the most useful of all applications, and that 92% of the PDA users surveyed used Epocrates [43]. Another survey of PDA usage by undergraduate medical students at the University of Alberta, Canada reported that 77.4% of students used drug reference applications, making them the most commonly used medical application in that population [53].

#### Medical Calculator Applications

A medical calculator or clinical calculator is a software program for calculating various clinical scores and indices such as body mass index (BMI), body surface area (BSA), coronary heart disease risk, individual drug dosing, etc. Usually calculation of clinical scores or indices involves complex formulas using several input parameters. Medical calculators typically provide a user interface to enter parameters and calculate scores using a standard formula. Users do not need to use or even know the actual formula for calculating a clinical score or index. For example, body mass index or BMI (also known as the Quetelet Index for its creator, Belgium statistician Adolphe Quetelet, 1796–1874), is the most commonly used measure of obesity internationally [54]. Our physician author has found that the $\frac{weight\left(kg\right)}{{\left(height(m)\right)}^{2}}$ or$\frac{weight\left(lb\right)\times 703}{{\left(height(in)\right)}^{2}}$ formula for calculating BMI, while simple to remember, is surprisingly time consuming and error-prone in the time-pressured environment of a busy clinic. The user only needs to enter a patient’s weight and height in a typical medical calculator, however, to calculate the BMI quickly and confidently.

Initially, medical calculator software programs were available on personal computers. Later, online versions of some calculators were accessible through the Internet. However, physicians were often unable to use this software at the point of care due to a lack of computer access. Now medical calculators are available for several smartphone platforms. Table 5 in Appendix II presents eight smartphone-based medical calculator applications that were discussed in 10 articles [11,16,26,39,41-43,47,55,56]. These eight applications are: Epocrates MedMath, MedCalc, Medical Calculator, Calculate, Archimedes, uBurn Lite, Softforce’s Antibiotic Dosage Calculator, and Paeds ED. The most commonly used medical calculators are MedMath and MedCalc [11,43]. The calculation of drug doses for pediatric patients is very crucial during medical emergencies, and the “Paeds ED” application calculates drug dosages for children based on their age in years [41]. Drug dosages for patients with renal failure can be calculated by Softforce’s Antibiotic Dosage Calculator. The uBurn app helps surgeons to calculate the percentage of total body surface area affected in adult burn victims [56].

#### Literature Search Applications

Literature search applications for healthcare professionals facilitate searching biomedical literature databases such as PubMed/MEDLINE, Essie, etc. to find and display medical reference information. The experience of our physician author is that these resources are seldom useful at the point of care and seldom used by clinicians in that setting. However, our review found six literature search applications discussed in 15 articles [41,42,45,50,51,55-64]. Table 6 in Appendix II presents the functions of these six applications, PubSearch, PubMed on Tap, MEDLINE Database on Tap (MD on Tap or MDoT), askMEDLINE, PICO, and Disease Associations.

The free PubSearch [51] and fee-base PubMed on Tap [41,42,56] are two commercial applications for the iOS platform that facilitate PubMed/MEDLINE search from iPhone. These two applications are client applications for PubMed/MEDLINE. The National Library of Medicine (NLM) provided MD on Tap (MDoT) to help healthcare professionals find answers to clinical questions and access medical reference information at the point of care through three search engines: PubMed, Essie, and Google [58]. In some articles [57,58], the NLM’s MDoT project was referred as “PubMed on Tap” since it was initially named as “PubMed on Tap”, which is different than the “PubMed on Tap” for iPhone mentioned at the beginning of this paragraph. MDoT is a client–server application in which the software client (available for Palm OS and Windows Mobile platform) sends the user’s plain-text query to the intermediate server (called MD on Tap server), which formats the query into appropriate search terms for the selected search engine (Essie, PubMed/MEDLINE, or Google) and returns the search results to the client application [58]. The search result is then displayed by grouping the articles into several clusters. The performance of MDoT in answering clinical question varies with the selection of search engine. Studies showed that Essie performed better than PubMed/MEDLINE [59,64]. However, later studies showed that medical residents using PubMed/MEDLINE on MDoT during daily rounds answered most of their clinical questions (68% and 86% in two separate studies) when queries consisted of three or more medical terms [63,64].

The MDoT client application required improvement in navigational and functional characteristics such as incorporating visual cues to indicate a visited citation [57]. The requirement of maintaining a high performance server for hundreds of concurrent users is a disadvantage for MDoT compared to client-only literature search applications like PubSearch or PubMed on Tap, since the search engines for these latter are already deployed in high performance servers and maintained by the providers. MDoT’s advantage is that the user interface was implemented independently while the processing power was delegated to the server and all transactions are stored in a local database on the server, facilitating research on users’ queries from mobile devices [58,64]. In 2007, the development and support for MDoT client application was stopped, introducing compatibility issues with future operating system versions [65].

Search results mainly depend on the query’s precision, so experienced users use specialized vocabulary such as MeSH to find relevant citations in PubMed/MEDLINE. The “PubMed for Handhelds” site (http://pubmedhh.nlm.nih.gov/) provides access to the PICO (Patient, Intervention, Comparison and Outcome), askMEDLINE, and Disease Associations (DA) search engines, developed by the NLM to facilitate literature search without knowing MeSH. PICO facilitates well-formatted search that includes four text fields: problem, intervention, compare to, and outcome [45,60,62]. The askMEDLINE search engine was developed from PICO to allow natural language query [45,60-62]. DA is a search interface for case reports and review of reported cases in PubMed/MEDLINE [45].

#### Clinical Communication Applications

Smartphones support several means of communication including voice calling, video calling, text messaging, email messaging, multimedia (text, image, and video) messaging, and conferencing through the cellular phone service provider. Besides standard communications, clinical communication applications are designed to simplify communication among clinicians within a hospital. A total of five articles discussing clinical communication using smartphones were included in this study [12,20,51,66,67]. Among them two articles discussed three smartphone-based communication applications [20,51], and three articles demonstrated the impact and improvement in clinical communication using smartphones [12,66,67].

Table 7 in Appendix II shows the functionality of three clinical communication applications that were discussed in two articles [20,51]. These three applications are Voalté One, Amcom Mobile Connect, and mVisum; all require the installation of proprietary communication servers. Voalté One combines phone calls, text messaging, and alarm alerts into one device. Amcom Mobile Connect is a messaging and alerting application that separates critical messages from less important one. mVisum is a specialized application for cardiology communications that receives monitor data, alarms, ECGs, and lab results on smartphones.

The use of mobile phone communication in critical care environments facilitates relaying important information quickly, reducing the risk of medical errors [66]. Nurses may need to go through a complex process to find the responsible physician for a patient using traditional numeric paging. In Toronto General Hospital, the use of smartphones simplifies reaching the responsible physician. Nurses can send email messages indicating priority and requesting feedback if necessary, or can call directly in emergency cases [12]. However, mobile communication may impact informal group discussions among workers in the common areas that support some important activities such as managing schedule changes, and discussions related to professional feedback and quality control. This impact in a surgical department was found to be negative and additional socio-technical mechanisms may be required to overcome this [67].

#### HIS Client Applications

Client applications for Hospital Information Systems (HISs), such as electronic health records (EHR), electronic medical records (EMR), and picture archiving and communication systems (PACS), provide the flexibility of accessing patient information securely from anywhere at any time. A total of five articles discussed the use of smartphones to access patients’ clinical information [20,22,23,51,68]. Table 8 in Appendix II provides detailed information about HIS client applications for smartphones. These applications provide some of the functionality of their PC counterparts. OsiriX Mobile is the client application for OsiriX PACS, which processes and displays images using the DICOM standard for digital image storage [22,23,51,68]. MEDITECH, a client application for MEDITECH EMR, and PatientKeeper Mobile Clinical Results, a client application for PatientKeeper EMR, provide access to patients’ clinical information from a hospital EMR including lab results, medication lists, clinical notes, problem lists, vital signs, and allergies [20]. AirStrip OB is designed for obstetricians to access their EMR’s real-time and historical waveforms, fetal strips and maternal contraction patterns.

#### Medical Training Applications

Smartphones are also used for medical training and continuing medical education (CME). CME provides training in the most current evidence-based medical practice [69]. A total of four articles discussed the use of smartphones in medical training [25,69-71]. Among them, one article discussed smartphone-based HIV/AIDS CME materials [25], another article evaluated print and smartphone-based CME for men’s sexual health, and the other two articles discussed two smartphone-based software applications for medical training. These two applications are listed in Table 9 in Appendix II: iCPR and iResus. Both of the applications are designed for the iOS platform and available for free. iCPR is a self-directed Cardiopulmonary Resuscitation (CPR) training application that is based on both American Heart Association and European Resuscitation Council guidelines. This application measures the chest compression rate and gives audiovisual feedback, improving the performance of chest compression by helping the user to achieve the correct chest compression rate [71]. The United Kingdom’s resuscitation guidelines including adult and pediatric algorithms are visualized in an interactive format in the iResus application. The use of iResus significantly improved the performance of certified advanced life support clinicians [70]. Both iCPR and iResus were evaluated in simulated clinical scenarios and further studies are required in real clinical settings [70,71].

Smartphone-based HIV/AIDS CME materials, including 3D learning scenarios simulating interactive clinical cases, were provided to the clinicians in urban and peri-urban clinics in Peru. This software, while not commercially available, gave flexibility to the mobile health care workers in accessing learning content in a resource-limited setting [25]. However, another study [69] showed no significant difference in evidence-based clinical choices between users of printed vs. electronic CME modules on men’s sexual health.

#### General Healthcare Applications

Seven smartphone-based applications were categorized as general healthcare applications, as they do not fit any of the categories discussed in the previous sections. These seven applications are HCSIT, Borboleta, LIFe-reader, Multimedia Paging Based Clinical Alarm, Outbreaks Near Me, H1N1 Swine Flu Update, and WISER. These applications were discussed in six articles [39,52,72-75]. Table 10 in Appendix II shows detailed information about these applications.

The Handheld Computer Smoking Intervention Tool (HCSIT) assists clinicians with smoking cessation counseling and improved physicians’ comfort level significantly in counseling patients about smoking cessation [72]. A real-time clinical alarm monitoring system was developed by van Ettinger et al. (2010) [75] to monitor intensive care unit patients by smartphone, displaying the alarms for an entire unit or one bed, with the vital signs at the moment of the alarm color-coded by severity. Outbreak Near Me provides real-time disease outbreak information and H1N1 Swine Flu Update provides a news feed for Swine Flu (H1N1) outbreaks [39]. H1N1 Swine Flu Update is now discontinued since the H1N1 pandemic has abated. WISER is an application for Emergency Medical Service Specialists that identifies chemical and biological hazards on the basis of symptoms and signs from NLM’s Hazardous Substances Data Bank (HSDB), and radiological and biological substance reports [74].

In homecare, nurses visit patients’ homes and may be isolated from the healthcare center. Borboleta, a smartphone-based mobile telehealth system, was developed in Brazil for nurses in primary healthcare to use during patient homecare visits. The nurses can complete the patient registration and schedule a visit on their smartphones instead of using paper and pencil, and the data is centrally stored in a web server. In addition, the nurses can access patient data, caregiver information, socioeconomic data, visit history, disease history, and medication history during homecare visits [73]. LIFe-reader, a CDSS system for nurses in geriatric homecare in Sweden, was implemented to help nurses to obtain patients medication profiles by scanning the European Article Number (EAN) on the drug package. As described in the section tittled “Drug Reference Applications”, it can also check for inappropriate drugs, drug-drug interactions, and therapeutic duplication. LIFe-reader reduces the drug-related risks of falling and drug-related admissions to hospitals [52].

### Applications for Medical and Nursing Students

There are many smartphone-based applications containing primarily as educational material for medical or nursing students. Seven articles discuss a total of eleven applications [11,17,23,41,47,51,56]. Table 11 in Appendix II lists the eleven applications and provides detailed information for each. They are I-Surgery Notebook, Eponyms, Netter’s Atlas of Human Anatomy, Netter’s Anatomy Flash Cards, Blausen Ear Atlas, Oxford Handbook of Clinical Specialties, Dissection, Cranial Nerves, iSilo, Mobipocket Reader, and Instant ECG.

The anatomy tools for students are very useful, and our physician author found the flash-card application to be a particularly good way to study during spare moments in medical school when books were not available. Netter’s Atlas of Human Anatomy contains more than 532 colored anatomic illustrations that are mainly designed for educational purpose [23,56] and its Netter’s Anatomy Flash Cards version contains 300 interactive flash cards [51,56]. Dissection is an anatomy tool that displays dissection of the human head and neck [41]. A set of ear-related video animations including cochlear implants, ear pressure, ear tubes, hearing loss, hearing tests, and otitis media are available in the Blausen Ear Atlas [41]. Cranial Nerves is a learning tool that includes interactive visualization along with textual information about cranial nerves and the skull base, based on high-resolution CT scans [41]. Instant ECG is a basic ECG tutorial application that includes ECG electrophysiology, myocardial action potential, associated waveforms, and intervals and segments [23].

The details of eponymous signs and diseases are available on smartphone through the Eponyms application [51]. Students as well as surgeons, surgical interns, and residents can use I-Surgery Notebook on their iPhone or Android phone during their surgical sessions to log surgical cases including procedure, pre-operative and post-operative diagnosis, list of involved surgeons, and type of anesthesia used [51]. The printed version of the Oxford handbook of Clinical Specialties, which includes 12 books, is available as handheld version on smartphones [41].

Several other medical books are available as electronic version through electronic book reader application on smartphone such as iSilo and Mobipocket Reader. iSilo is a fee-based program that stores text in a highly compressed format and facilitates text search within a document or set of documents [17,47]. Mobipocket Reader is available for free and includes a library of all eBooks stored in local media, with the ability to annotate, highlight, or bookmark any part of the eBook, and lookup any word in several dictionaries [11].

Smartphone-based healthcare applications for clinicians, discussed in the section titled “Application for Healthcare Professionals”, can also be used by medical or nursing students for educational purposes. For example, Cibulka & Crane-Wider (2010) [76] designed teaching strategies for nursing students using smartphones that includeed a clinical consult guide, a prescribing reference, and a differential diagnosis tool from Skyscape or Epocrates. The students used the software packages during their classes to access clinically relevant information and found very useful. In a systematic review by Kho et al. (2006) [77], medical calculators and drug reference applications were also found to be very useful in medical education.

### Applications for Patients

Fourteen articles discuss fifteen smartphone-based patient oriented applications [23,29,32,35,41,78-86]. Table 12 in Appendix II shows detailed information about these applications. There are six applications for management of chronic conditions: Diabeo, Cardiomobile, Pulmonary Rehabilitation, PAL Calculator, Asthma Peak Flow Monitoring, and eCAALYX; four ENT-related applications including Hearing Check, uHear, iTinnitus, and Sleep Aid; three applications for human fall detection including Fall Detector by Hansen et al. (2005) [78], Fall Detector by Zhang et al. (2006) [80], and iFall; and two others: Purdue Momentary Assessment Tool, and Mayo Clinic Meditation.

Chronic disease management applications provide expert feedback to patients based on their input. The Diabeo system helps diabetic patients by calculating bolus insulin dose based on carbohydrate intake, pre-meal blood glucose, and anticipated physical activity reported; and automatically adjusts carbohydrate ratio and basal insulin. The patient data is sent from a smartphone through a General Packet Radio Service (GPRS) connection to a medical staff computer and stored there to facilitate further tele-consultation. The Diabeo telemedicine system reduces the cost of care and improves metabolic control for diabetes patients [32].

Cardiomobile is a real-time remote monitoring system for exercise-based cardiac rehabilitation that is comprised of a heart and activity monitor, single lead ECG, GPS receiver, and programmed smartphone (i-Mate SP3, Dubai). The GPS receiver and monitor connect to a smartphone via Bluetooth. The smartphone sends ECG rate, walking speed, heart rate, elapsed distance, and patient location to a secure server for real-time monitoring by a qualified exercise scientist. The system supports cardiac patients who are unable to access hospital-based rehabilitation due to excessive travel time or lack of available programs in their area [35].

Pulmonary Rehabilitation is an application for chronic obstructive pulmonary disease (COPD) rehabilitation and self-management, developed for smartphones by Marshall et al. (2008) [29], using a Bluetooth pulse oximeter to measure the heart rate during exercise. Patients can follow the step-by-step exercise instructions on their smartphones displaying patient heart rate, exercise time remaining in seconds, and feedback color (green: normal physical condition, amber: normal condition but near acceptable limits, red: dangerous physical condition). No assessment or clinical evaluation of this application was reported [29].

The measurement of physical activity level (PAL) is important in many chronic diseases. The PAL Calculator application uses smartphone questionnaires to calculate PAL. Study results demonstrated a high accuracy from this method in comparison to reference values [82].

Ryan et al. (2005) [79] described an observational study on asthma peak-flow monitoring using an electronic peak-flow meter connected to a smartphone. The application sends peak-flow readings through the GPRS network to a secure server, and receives asthma trend analysis feedback from the server. The study demonstrated a satisfactory primary outcome measure [79]. eCAALYX is a remote monitoring system for elderly patients with multiple chronic conditions presented by Boulos et al. (2011) [83] that receives data from wearable health sensors in a *smart garment*, transmits data to the monitoring server, and identifies higher-level information such as tachycardia and signs of respiratory infections based on established medical knowledge. Users can see current medical reports on their smartphones based on sensor data, perform new measurements, and communicate with caregivers through the application [83].

Hearing Check and uHear are two free hearing loss self-assessment tests for iPhone. Hearing Check was developed by the UK Royal National Institute for Deaf People (RNID), and calls a landline number to receive a free hearing check [86]. uHear includes three assessment tests: hearing sensitivity, speech in noise, and a questionnaire about common listening situations [41]. In addition to its use as a self-testing device, our physician author has used uHear in low-resource clinical environments where complete audiometry was not available. iTinnitus is a sound-therapy application for patient with tinnitus, which records tinnitus by frequency in Hertz and keeps track of the tinnitus in a daily diary graph. iTinnitus supports full masking therapy with a sound played at a frequency around the same frequency as the patient’s tinnitus [41]. Sleep Aid is a sleep apnea management application that records snoring, generates a graph of the snoring, plays the recorded snoring sound, and provides information about sleep apnea [41].

Three human fall detection applications were prototyped using smartphones. Hansen et al. (2005) [78] used a separate wearable tri-axial accelerometer and camera phone to build a fall detection system. The accelerometer processes the data locally that identifies a fall and send the data to the phone during a suspected fall. The phone then requests vocal or keypad response from the user, and if there is no response it automatically makes an emergency call and sends data and video from the phone’s camera to the emergency system [78]. The user may forget to wear the sensor, however, which remains a major problem with fall detection systems based on a wearable accelerometer. Zhang et al. (2006) [80] embedded the tri-axial accelerometer in the cell phone to overcome the problem. In cases of possible falls, the data is sent to the server for further analysis. This has been shown to be an effective method for fall detection [80]. Sposaro and Tyson (2009) [81] demonstrated the cost-effectiveness of iFall, which utilizes the inbuilt tri-axial accelerometer of an Android phone, processes the data on the phone, and prompts the user in cases of possible falls. If the fall is confirmed by the user not responding to the application, iFall makes an emergency call [81].

The Purdue Momentary Assessment Tool (PMAT) is a human behavior monitoring tool using smartphones to facilitate event-driven study design [87]. PMAT was successfully used to monitor substance use and symptom expression in schizophrenia patients [84,85]. Mayo Clinic Meditation helps patients practice meditation with a short training video introducing the key concepts of meditation, and 15-minute and 5-minute meditation programs [23].

### Application Distribution

**Figure 2:**
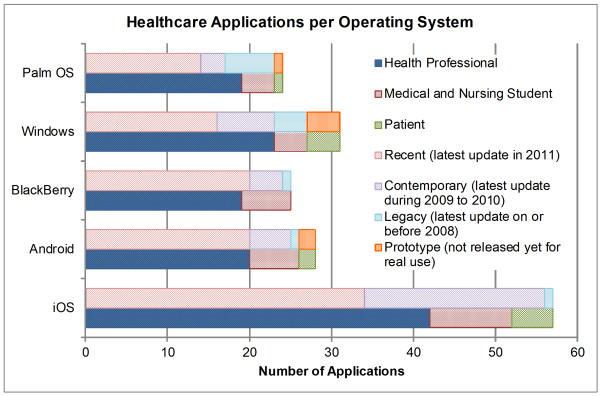
**Number of Healthcare Applications per OS Platform Discussed in this Study.** This figure presents the distribution of the smartphone-based healthcare applications that are discussed in this study for all of the six major OS platforms. The distribution is describes in two categories: the first breakdown is according to the intended users, that is, healthcare professionals, medical and nursing students, and patients; and the second breakdown is according to their latest release or update date, that is, recent (latest update in 2011), contemporary (latest update during 2009 to 2010), legacy (latest update on or before 2008), or prototype (not released yet for real use).

In this study, a total of 83 applications were discussed. Among them, 57 applications were designed for clinicians, 11 applications were designed for medical or nursing students, and 15 applications were designed to be used by the patients. Figure 2 presents the distribution of these applications for the major smartphone OS platforms. 74 applications are developed for at least one OS platform and the remaining 9 applications can be accessed either on a web-enabled or java-enabled smartphone. None of the six OS platforms (discussed in Appendix I) support all of these 74 applications. iOS is the most popular platform for healthcare smartphone applications because 57 out of 74 applications are developed for iOS.

Figure 2 also presents a breakdown of the number of applications in two categories: (1) intended users (healthcare professionals, medical and nursing students, and patients), and (2) their latest release or update date (recent (latest update in 2011), contemporary (latest update during 2009 to 2010), legacy (latest update on or before 2008), or prototype (not released yet for real use)). Most of the applications for each platform are intended for healthcare professionals. For example, 74% of the iOS applications and 71% of the Android applications are for healthcare professionals. The percentage of applications for medical and nursing students for each OS platform is below 25%. The Windows platform has the highest number of applications (12.9%) for patients and BlackBerry has no application for patients. At least half of the applications have an updated or new version release in 2011. The BlackBerry has the highest ratio (80%) of recently released application followed by 71.4% for Android and 59.6% for iOS. The iOS has the highest number of applications but a large portion (38.6%) of the applications was released in 2009 and 2010. The Palm OS has the least number of total applications but has the highest ratio of legacy (25%) applications.

Symbian OS has a very low coverage of healthcare applications because only three applications are developed for the OS. Other five OS platforms (i.e. iOS, Android, Palm, Windows, and BlackBerry) have extensive coverage of healthcare applications because at least one application is available for all of these five OS platforms in each of the broader category. In the following, the coverage of healthcare applications for these five OS platforms is discussed.

In “Disease Diagnosis” category, we discussed a total of nine medical reference applications (i.e. JHABx, 5MCC, 5MIDC, SG, ePocrates ID, ID Notes, PMID, and UpToDate) in which at least four of them are available for all of these five OS platforms. We also discussed six lab reference applications (i.e. Palm Lab DX, Normal Lab Values, Lab Unit Converter, Labs 360^0^, Davis’s Laboratory and Diagnostic Tests, and Pocket Guide to Diagnostic Tests) in the same category in which at least three of them are available for all of these five OS platforms. There are six other useful “Disease Diagnosis” applications (including Eroentgen Radiology Dx, iSeismometer, Video Laser Level, EyeChart, EyePhone, and DizzyFix) that are available for iOS only except iSeismometer that is also available for Windows Phone.

For the six “Drug Reference” applications, at least two of them are available for all of these five OS platforms. Among the nine “Medical Calculator” applications, three specialist medical calculators (i.e. uBurn Lite, Softforce’s Antibiotic Dosage Calculator, and Paeds ED) are available for iOS only and at least two out of six other general purpose medical calculators are available for all of these five OS platforms. The “Literature Search” applications are mainly developed for iOS, Palm and Windows Phone platforms. However, three applications for literature search can be accessed from any web-enabled smartphone through their native internet browser. Accordingly, the “Literature Search” applications are available virtually for any OS platforms. The “Clinical Communication” and “HIS Client” applications are not available for all OS platforms. These applications require installation of proprietary servers and the vendors of these servers produce the smartphone applications based on their clients’ requirements. Currently, clinical communication and HIS client applications are mainly available for iOS, Android, and BlackBerry. The “Medical Training” applications are available for iOS only. The “General Healthcare” applications, “Medical Education” applications, and applications for patients do not serve a common purpose in their category. In order for these applications to be widely used in practice, they should be available for all OS platforms. Every medical education application except “MobiPocket Reader” is available for iOS. Not a single application in the category of “Application for Patients” is available for two different OS platforms.

Using multiple smartphone devices for use of several healthcare applications is not practical for all three user groups including healthcare professionals, medical and nursing students, and patients. We believe every healthcare application should be developed for at least the top 2 to 3 OS platforms. We can observe that there is low coverage in the following categories: specialist medical calculator, clinical communication, HIS client, medical training, general healthcare, medical education and applications for patients. The future development should focus on increasing the coverage of applications for all OS platforms in the above mentioned areas.

## Discussion

Smartphones access clinical applications, evidence-based resources, and advanced mobile communication in one handheld-sized device at the point of care. Their mobility enables health professionals to use them in a clinical setting for patient care [88]. This study presents smartphone-based healthcare applications that were discussed in the literature. The applications were categorized based on target users: clinicians, medical or nursing students, or patients. The functionalities of the applications and supported smartphone platforms were discussed and presented in tabular format.

Studies show wide adoption of smartphones by healthcare professionals during recent years [1,2]. Smartphones are becoming popular for clinical use among clinicians, and medical and nursing students [43,53,55,77,89-91]. PDAs, the predecessors of smartphones, have been recognized in a systematic review article as useful in physicians’ practices for rapid response, error prevention, and data management and accessibility [92]. Real-time clinical information at the point of care is very important in the practice of EBM, since clinicians may not seek answers to clinical questions after completion of a clinical encounter [93,94]. Drug reference applications, medical textbooks and references for disease diagnosis, and medical calculator applications were reported as the most useful by clinicians and medical and nursing students [43,53,77,89,91,95]. Single applications may not provide all required information, requiring use of combinations of applications [40].

Medical reference applications, such as the Sanford Guide, Johns Hopkins Antibiotic Guide, 5-Minute Infectious Diseases Consult, 5-Minute Clinical Consult, and ePocrates ID, provide treatment recommendations for most cases [46,47]. UpToDate is a useful evidence-based clinical information tool at the point of care, but requires an institutional or personal annual subscription [16,17,40,45]. The drug-drug interaction-checking feature of drug reference applications like Epocrates and Skyscape’s RxDrugs is a very useful evidence-based resource at the point of care [11,40]. Medical calculators help clinicians calculate various clinical scores and indices, and MedMath and MedCalc were reported as the most commonly used among eight medical calculators listed in Table 5 [11,43].

The literature search applications listed in Table 5 facilitate PubMed/MEDLINE search from smartphones. The use of a specialized medical vocabulary (such as MeSH) in search queries is very effective for finding relevant citations. Usually, queries consisting of three or more medical terms can retrieve relevant documents to answer clinical questions successfully [63,64]. However, learning effective search strategies using a specialized vocabulary could be impractical for clinicians and the general public [96,97]. Therefore, the NLM developed smartphone-based applications like askMEDLINE, PICO, and Disease Associations for non-expert clinical information seekers [45,60-62]. The presentation of search results for clinical use on the small screens of smartphones is challenging. The visualization of the search result by grouping or clustering retrieved documents, with summary information for the group, was found to be useful for effective and intuitive navigation [24,58,98-100].

The use of smartphones for mobile clinical communication facilitates various means of communication among clinicians, such as text messages, email messages, voice, video, and images, and reduces communication delay [12,20,66]. However, mobile communications in clinical settings may have a negative impact on informal interaction among workers and require socio-technical mechanisms to overcome this [67]. Bedside access, or access at anytime from anywhere, to patients’ clinical information from Hospital Information Systems (HISs) can be facilitated through smartphones [20,22,23,51,68]. Also, electronic capture of patient data into HIS within resource-limited settings in developing countries has become feasible, using smartphone-based applications [101]. However, HIS client applications must comply with national legislation on privacy and security, such as the U.S. Health Information Portability and Accountability Act [102], the European Union’s regulation 95/46/EC on processing of personal data [103] or the UK National Health Service’s privacy and confidentiality issues [104], etc. [33,51,55,105,106].

Medical training applications on smartphones make medical guidelines or CME materials accessible from anywhere, including resource-limited settings [25,69-71]. Current disease outbreak updates, real-time ICU patient monitoring, and electronic mobile homecare have also become feasible as a result of smartphone technology [39,52,73,75]. Handheld versions of medical books, journals like BMJ and JACC, interactive anatomy tools, medical calculators, medical references, and drug references on smartphones provide mobile learning opportunities for medical and nursing students [11,17,23,41,47,51,56,77,107-109]. Accordingly, these applications may be included in medical or nursing curriculum [76,110].

There are many advantages of using smartphone-based healthcare applications in medical practice. For example, they allow for advanced mobile clinical communications using multimedia functions and provide access to various clinical resources at the point of care such as up-to-date evidence-based clinical resources, medical formula calculator, drug reference and interaction checking, etc. In addition, they can provide secure remote access to real-time patient monitoring system and EMR systems for better patient care. Hospitals should encourage healthcare professionals to use smartphone-based healthcare applications, and financially support them to keep the applications up-to-date with regular update.

Smartphone-based patient oriented applications deliver healthcare services for patients with chronic conditions, which is the purpose of mobile health or mHealth [29,32,35,79,82,83]. The mHealth component of eHealth delivers medical and healthcare services through mobile devices [111]. The World Health Organization (WHO) has recently defined mHealth as “medical and public health practice supported by mobile devices, such as mobile phones, patient monitoring devices, personal digital assistants (PDAs), and other wireless devices” [112]. The wide adoption of high-functionality smartphones by the general public highlights the increased demand for better mHealth services through smartphones [3-6,86]. Mobile telemedicine services with video capability have become viable by using smartphones [30,33]. However, while the advancement in smartphone-based mHealth services may be seen in developed countries, developing countries have yet to get its potential benefits [42]. It is anticipated that about 500 million smartphone users around the world will use mHealth services by 2015 [113]. Since anyone can implement applications for smartphones, healthcare applications need to be controlled and validated through appropriate organizations such as the U.S. Food and Drug Administration (FDA), the Australian Therapeutic Goods Administration (TGA), the International Federation of Clinical Chemistry (IFCC), etc. [19,26,114]. For example, Abroms et al. (2011) [115] examined 47 iPhone applications for smoking cessation that were found to have low adherence to clinical practice guidelines.

An interesting feature of smartphone devices is Bluetooth, which is a technology for short-distance wireless data transmission. Nowadays, many medical devices (such as glucose meters, thermometers, etc.) have this functionality. In order to standardize the interoperability between medical devices using Bluetooth, the medical working group of the Bluetooth Special Interest Group provided the specification for Bluetooth Health Device Profile (HDP) [116]. The smartphones should support built-in Bluetooth HDP for standard Bluetooth communication with medical devices. This will enable the smartphone applications to work with medical devices from different vendors. Currently, only the Android platform supports built-in Bluetooth HDP (see Appendix I).

The challenges of smartphone-based healthcare include limited battery life, small screen size, potentially erroneous data input, computer viruses including spyware, magnetic interference with medical devices, potentially inefficient patient-physician interactions, loss or theft, and breaches of data privacy and security. Data input is much slower and erroneous on smartphones using a stylus [117]. Antivirus software must be used to protect devices from computer viruses and spyware, and must be updated regularly. The use of smartphones in hospital environments may have a small risk of electromagnetic interference with medical devices and they should be used with caution in certain areas. Studies suggest that using mobile devices in a normal way beyond a one-meter range of medical devices is safe [66,118-121]. The interaction between patients and physicians may be hampered by the use of a PDA during the patient encounter, but explaining the reason for using a PDA to the patient was found to have a positive effect on patient-physician interactions and communications [122]. The privacy and security concerns of storing or communicating patient data with smartphones should be addressed cautiously. These security features of smartphones, while not available for all devices, may be useful: data backup, encryption of stored patient data, remote wiping to destroy all data on a device in case of loss or theft, and securely encrypted wireless data transmission over WiFi [123-126].

The information contained in healthcare applications must be accurate. In general, application users must agree with the terms and conditions of use of applications to use the applications, and the users are mainly liable for utilizing the information in the applications. As a result, incorrect or outdated information from healthcare applications may lead to medico-legal consequences and users instead of software companies are responsible for them. This problem may affect many healthcare applications including disease diagnosis, drug reference, and medical calculator applications. The peer reviewed applications (such as JHABx guide) are more reliable than non-peer reviewed applications [47]. There are a few articles that discuss the accuracy of some selected applications [47,127]. Our future work is to provide guidelines for developing and using smartphone-based healthcare applications in medical practices. The focus would be on medico-legal and ethical issues regarding use of smartphone-based healthcare applications.

### Limitations

Many smartphone-based medical applications are available in online application stores (e.g., Apple’s App Store). However, most of them have not been discussed in the medical literature. Those healthcare applications were not included in this study. We would like to emphasize that our goal was to systematically review articles in the academic literature discussing smartphone-based healthcare applications.

## Conclusions

In this study, we discussed many smartphone-based healthcare applications from the literature. These applications were grouped according to targeted users (i.e., clinicians, medical and nursing students, and patients). These applications are not intended to replace desktop applications, but to add to existing technologies for better healthcare. The functionalities of the applications are growing day by day and new functionalities are available with every major release. The work of healthcare professionals is very mobile in nature. Smartphones enable for advanced mobile communication between health professionals, makes medical formula calculations available anywhere anytime, and provides access to evidence-based medical resources including disease diagnosis guides, drug references, literature search, and continuing medical education materials at the point of care. In addition, smartphones enable health professionals to access to EMR systems from anywhere thus facilitating remote consultation and telemedicine. Moreover, performing simple medical exams such as visual acuity test is also viable using a smartphone. The wide adoption of smartphones by the general public emphasizes the opportunity of better mHealth and mobile telemedicine services through patient oriented applications, for example, patient education, disease self-management, and remote monitoring of patients.

Most of the applications discussed in the study are standalone applications. There is an immense need for developing guidelines for standardizing smartphone-based healthcare applications so that the applications are used together seamlessly for specific purposes and are integrated with HISs such as EMR and patient monitoring systems to maximize the power of mobile applications. This will enable healthcare professionals to use the applications in a more meaningful way for better patient care.

Smartphone-based applications are getting more attention in healthcare day by day; all of the 55 articles included in this systematic review were published after 2003 and 24 of these articles were published recently between January 2010 and April 2011. The full potential of smartphones has yet to be exploited. We believe that this study provides a better understanding and greater insight into the effectiveness of the smartphone-based healthcare applications in improving patient care and reducing healthcare expenses.

## Appendix A

### Appendix I: Smartphone Platform Overview

**Table 1:** Smartphone Operating-System Platforms, OS Features

**OS Platform →**	**Symbian OS**[158]	**Palm Web OS**[159,160]	**Windows Phone**[161]	**BlackBerry**[162,163]	**iOS**[164-166]	**Android**[140,167]
**Version**	3	2.3	7	6	5	4.0
**App Store**	Ovi Store	Palm App Catalog	Marketplace	BlackBerry App World	App Store	Android Market
**Developer**	Nokia	Hewlett Packard (HP)	Microsoft Corporation	Research In Motion (RIM)	Apple INC.	Google INC.
***OS Feature***						
**Multitasking**	✓	✓	✓^l^	✓	✓	✓
**Notifications**	✓	✓	✓	✓	✓	✓
**System Bar**	✓	✓	✓	✓	✓	✓
**Tool Bar**	✓	✓	✓	✓	✓	✓
**Customizable Home Screen**	✓	×	✓	✓	✓	✓
**Recent Apps**	×	×	×	×	✓	✓
**Text Selection, Copy & Paste**	✓	✓	✓	✓	✓	✓
**App folders**	✓	×	×	✓	✓	✓
**Universal Search**	×	✓	✓	✓	✓	✓
**Adobe Flash**	✓	✓	✓	✓	×	✓
**Live Streaming**	RTSP	RTSP	RTSP	RTSP	HTTP	HTTP, RTSP
**Widgets**	✓	×	✓	×	×	✓
**Encryption**	✓^*^	×	✓	✓	✓	✓^*^
**Remote Wipe**	×	✓	✓	✓	✓	✓
**WiFi Security**	WEP, WPA, WPA2	WEP, WPA, WPA2	WEP, WPA, WPA2	WEP, WPA, WPA2	WEP, WPA, WPA2	WEP, WPA, WPA2
**Multilingual**	✓	✓	✓	✓	✓	✓
**Accessibility**	✓	✓	✓	✓	✓	✓

This table compares the operating-system features of six smartphone platforms: Symbian OS, Plam Web OS, Windows Phone, BlackBerry, iOS, and Android.

✓ This feature is supported by the OS platform.

× This feature is not supported by the OS platform.

✓^*^ This feature is supported in a limited number of devices.

✓^l^ The functionality presented by this feature is limited.

**Table 2:** Smartphone Operating-System Platforms, Features Support with Hardware

**OS Platform →**	**Symbian OS**[158]	**Palm Web OS**[159,160]	**Windows Phone**[161]	**BlackBerry**[162,163]	**iOS**[164-166]	**Android**[140,167]
** Touch screen**	✓^*^	✓	✓	✓	✓	✓
**Multi-touch User Interface**	✓	✓	✓	✓	✓	✓
**Virtual Keyboard**	✓	✓	✓	✓	✓	✓
**External Keyboard**	✓	✓	✓	✓	✓	✓
**Camera**	✓	✓	✓	✓	✓	✓
**Video Recording**	✓	✓	✓	✓	✓	✓
**Video Calling**	×	×	×	×	✓^*^	✓^*^
**USB Interface**	✓	✓	✓	✓	✓	✓
**Voice Command**	✓	×	✓	✓	✓	✓
**Tethering**	✓	✓	×	✓	✓	✓
**Multicore Processor Support**	×	✓^*^	×	×	✓^*^	✓^*^
**Accelerometer**	✓^*^	✓	✓	✓	✓	✓
**Gyroscope**	×	×	✓	×	✓	✓
**Switch Screen Orientation**	✓	✓	✓	✓^*^	✓	✓
**GPS**	✓	✓	✓	✓	✓	✓
**Bluetooth**	✓	✓	✓	✓	✓	✓
**Built-in Bluetooth Health Device Profile**	×	×	×	×	×	✓
**WiFi**	✓	✓	✓	✓	✓	✓
**3G/4G**	3 G	4 G	3 G	3 G	4 G	4 G

This table compares the support of features by six smartphone OS platforms with the availability of hardware in the device.

✓ This feature is supported by the OS platform.

× This feature is not supported by the OS platform.

✓^*^ This feature is supported in a limited number of devices.

#### Smartphones

Smartphones are essentially cell phones with advanced connectivity and computing capability. There is no standard definition of smartphone found in the industry. In the annual report published in 2010 by the U.S. Federal Communications Commission (FCC), smartphones are defined as mobile devices with cell-phone capability having “an HTML browser that allows easy access to the full, open Internet; an operating system that provides a standardized interface and platform for application developers; …a larger screen size than a traditional handset…and touch screens and/or a QWERTY keypad” [128]. Another report submitted to the FCC by the Telecommunication Industry Association (TIA) in 2010 defined a smartphone as “a mobile device that offers the most advanced compu-ting ability and connectivity available today….having intelligence similar to personal computers while offering the capabilities of a mobile phone…running robust operating systems (OS) software that provides platforms for…third-party applications….having more processing power and memory…multiple connections, such as WiFi and Bluetooth, multimedia applications, such as photos, music, and video, and GPS functions” [129].

The first commercial cell phone was released publicly in 1983, 10 years after a cell phone prototype was first publicly demonstrated in April 3, 1973 with a call placed by Martin Cooper, the general manager of Communications Systems division at Motorola [130]. Since then, the development of cell phone technology has advanced very quickly due to the huge demand. The advancement of cell phone technology includes more powerful microprocessors, larger screen, and longer battery life. In a parallel trend, handheld-sized computers or Personal Digital Assistants (PDAs) emerged in the early 1990’s with the advancement of computer technology. Most recently, cell-phone capability has been introduced in PDAs, reducing the need to carry two separate devices, and producing the so-called smartphone. Thus, smartphones are the convergent technology of cell phones and PDAs [131]. In addition to phone services (voice calling, text and multimedia messaging, etc.), the common functionality of smartphones includes e-mails, calendars, contact lists, task lists, camera and video capabilities, and Internet access [132]. Smartphones are also generally equipped with Bluetooth, WiFi and USB connectivity.

#### Smartphone Platforms

**Figure 3:**
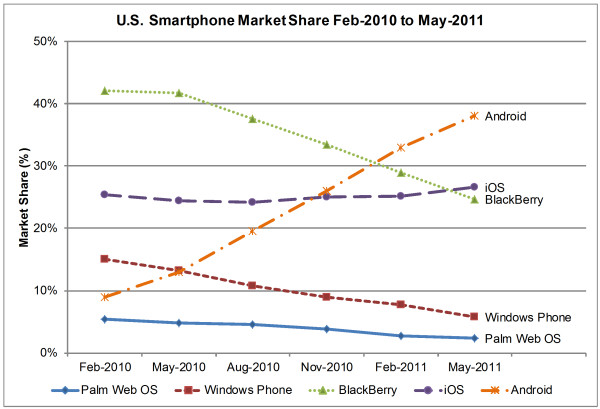
**Smartphone U.S. Market Share Feb-2010 to May-2011**[3-5]**.** This figure presents the market share of five major smartphone platforms (i.e. Palm Web OS, Windows Phone, BlackBerry, iOS, Android) in the United States during the period of 2010 – 2011. In the middle of 2011, Android has become the leader in smartphone market share while all other platforms have shown decreasing trend except iOS. The market share of iOS was almost consistent during this period.

**Figure 4:**
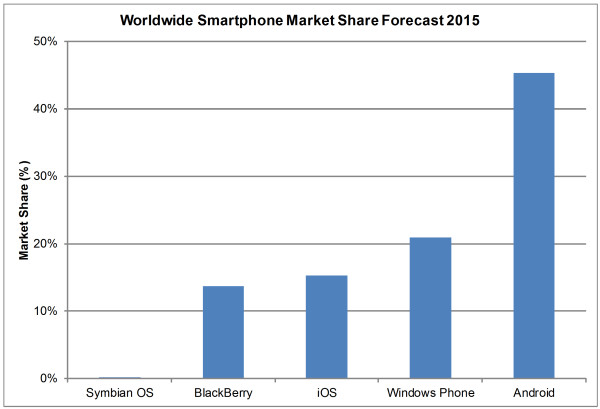
**Smartphone Worldwide Market Share Forecast 2015**[133]**.** This histogram presents the worldwide market share forecast data from IDC for six smartphone OS platforms in 2015. Android is predicted to be the global market leader in smartphones acquiring almost half of the market share by 2015.

**Figure 5:**
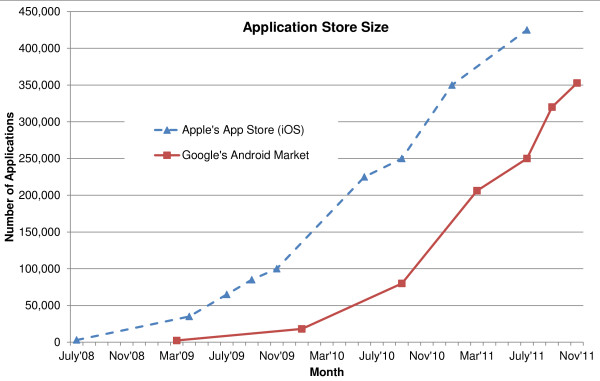
**Smartphone Worldwide Market Share Forecast 2010–2015**[134]**.** This figure presents the market share forecast data from Gartner for five smartphone OS platforms up to 2015. Android is predicted to be the leader in smartphone market by 2015 acquiring almost half of the market share. Symbian OS will lose almost all of the market share since its vendor Nokia announced in February, 2011 to shift from Symbian OS to Windows Phone 7 [153], thus global market share for Windows Phone is forecast to gain by 2015, placing the platform in second position.

Smartphones are usually based on specially designed operating system (OS) platforms for mobile computing and phone services. These OS platforms are now capable of running third-party applications including medical and healthcare applications, which is a great advantage of smartphones [26]. There are six major smartphone OS platforms: Symbian OS, Palm OS, Windows Phone, BlackBerry, iOS, and Android. Figure 3 illustrates three-month market-share averages for different smartphone OS platforms in the U.S. from February 2010 to May 2011 [3-5]. Figures 4 and 5 illustrate worldwide market share forecasts of smartphone platforms through 2015, published during the first quarter of 2011 by IDC [133] and Gartner [134] respectively. 

An overview of OS features of smartphone platforms is illustrated in Table 1. All of the platforms provide standard applications such as organizers, contact lists, e-mail, Web browsers, photo galleries etc. The *multitasking* feature allows multiple applications to run concurrently. The *notification* system bar displays system status (e.g. battery, network, etc.) and notification (e.g. received text messages, email messages, etc.) while the user works on an application window, and the *toolbar* facilitates control buttons (e.g. maximize, minimize, close) for applications. In some smartphones, the home screen is *customizable* to allow the user to add or remove application shortcuts in the home screen, and also allows the user to drop *widgets* onto the home screen. The *app folder* facilitates storing applications in a special system-defined folder, and all recently used applications are displayed in *recent apps*. Smartphones now support text selection, copying to the clipboard, and pasting anywhere. The search box may combine searching of the Web and the device internally together, which is called *universal search*. The *Adobe Flash support* is very attractive to the user as it allows all the flash based resources (e.g. games, videos etc.) from the Web. HTTP or RTSP live streaming facilitates real-time audio or video streaming. Data security can be enhanced by storage area *encryption*, *remote wiping* (during loss or theft), and *WiFi security* for secure wireless data transmission. Some platforms also support accessibility features for the disabled [129,135-138] and multiple languages.

Table 2 illustrates the support of common features by smartphone OS platforms with the availability of hardware in the device. These are touch screens, multi-touch user interfaces, virtual keyboards, external keyboards, cameras, video recording, voice commands, tethering (Internet-connection sharing with other devices using cable, Bluetooth, WiFi, etc.), multi-core processor support, accelerometers, gyroscopes, screen re-orientation, Global Positioning System (GPS) features, Universal Serial Bus (USB) connections, and wireless connections (Bluetooth, WiFi, 3G/4G).

#### Symbian OS

Symbian OS was developed by Symbian Ltd. and subsequently acquired by Nokia. The most recent release of this platform is Version 3 as of June 2011. This platform is prominent globally but not in the U.S. [6,134]. Symbian OS had nearly 40% of global market share in 2010 but was forecast to be below 1% by 2015 (Figures 4 and 5), which may be due to Nokia’s February 2011 announcement to shift from Symbian OS to Windows Phone 7 [133,134,139]. Symbian OS supports almost every feature listed in Tables 1 and 2 except recent apps, universal search (it supports internal search only), remote wiping, video calling (third-party software may be available), multi-core processor support, and gyroscopes. Stored-data encryption and accelerometers are available on selected devices only.

#### Palm Web OS

Palm Web OS (Version 2.3 as of June 2011) is the successor of Palm OS, which was introduced in January 2009 by Palm and acquired by Hewlett Packard (HP) in February 2011. Palm has a small market share in the U.S. with a decreasing trend during 2010–2011 (See Figure 3). Palm supports almost all the features listed in Tables 1 and 2 except recent apps, app folders, widgets, storage-area encryption, video calling, voice commands, and gyroscopes. Palm provides multi-core processor support but this is currently available in touch-pad devices only, not in smartphone devices.

#### Windows Phone

Windows Phone (Version 7 as of June 2011) is the successor of the Windows CE and Windows Mobile platforms developed by Microsoft. During 2010–2011, Windows Phone lost about 9.3% of U.S. market share within a 15-month period (Figure 3); however, global market share is forecast to gain by 2015, placing the platform in second position (Figures 4 and 5). Nokia announced in February 2011 a switch from Symbian OS to Windows Phone [139]. The multitasking support in Windows Phone is restricted to allowing third-party applications to run limited actions in the background. Unsupported features are recent apps, app folders (folders are arranged in hubs), support for external keyboard, video calling, tethering, and multi-core processor support. Windows phones provide a hardware button to facilitate universal search.

#### BlackBerry

BlackBerry (Version 6 as of June 2011) was developed by Research In Motion (RIM) of Canada and is very prominent in the U.S. BlackBerry lost 17.4% of U.S. market share in a 15-month period during 2010–2011 (Figure 3), but its global market share was forecast to decrease little before 2015 (Figures 4 and 5). BlackBerry Version 6 is only available in newly released BlackBerry devices, and devices with old platforms cannot be upgraded to version 6. This platform does not support recent apps, widgets, external keyboards, video calling, tethering, or multi-core processor support. The remote wiping feature is available through the *BlackBerry Protect* application, which is available free of cost.

#### iOS

iOS (Version 5 as of November 2011) was developed by Apple for their iPhone (the only smartphone based on iOS), iPod, and iPad. Its 2010–2011 U.S. market share was consistently around 25%, placing it in second position at the end of May 2011 (Figure 3). The global market share forecast is also consistent through 2015 with some fluctuations, remaining in third position by 2015 (Figures 4 and 5). This is the only platform among the six platforms discussed in this article that does not support the popular *Adobe Flash Player*. There is also no support for installing widgets, and multi-core processor support is available only on the iPad. The video calling functionality is available using the FaceTime application (developed by Apple) in iPhone 4, iPod, and iPad. This platform is very prominent for its user interface and multi-touch gesture functionality. Unlike other platforms, notifications are not displayed in the system bar and the user needs to slide down from the top to access notifications. The iPhone is only smartphone device available based on iOS, and no future plans have been announced to release others.

#### Android

Android (Version 4.0 as of November 2011) is an open-source platform that was initially developed by Android and later purchased by Google. This platform is becoming prominent in the U.S. as well as globally [6,133,134]. Android U.S. market share increased from 9% to 38.1% within a 15-month period during 2010–2011, placing this platform in the top position (Figure 3). Android is predicted to be the global market leader in smartphones, acquiring nearly 50% market share by 2015 (Figures 4 and 5). This platform supports all the features listed in Tables 1 and 2, but some features (storage-area encryption, video calling, and multi-core processor support) are available in selected smartphone devices only. The system bar provides a software navigation button in addition to system status and notifications. Of the six major OS platforms, only Android 4.0 has built-in support for connecting to Bluetooth Health Device Profile (HDP) devices [140].

#### Smartphone Applications

**Figure 6:**
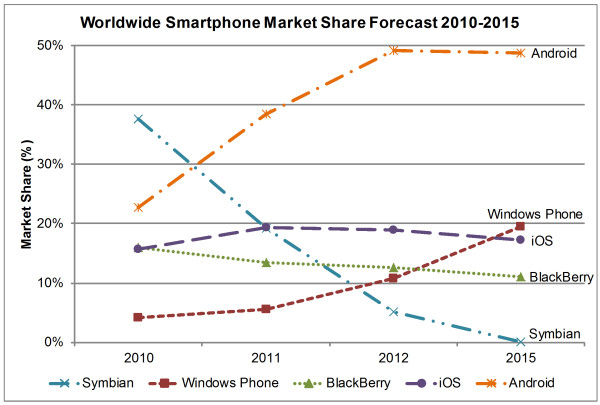
**Number of Applications in Apple’s App Store and Google’s Android Market (July’08 – Nov’11)**[141-156]**.** This figure presents the growth rate of two major smartphone application stores: Apples’s App Store and Google’s Android Market, during the period of July, 2008 to November 2011. Both of the stores are growing very fast. According to the most recent updates, the total number of applications in Apple’s App Store is more than 425,000 as of July, 2011 [141] and in Android Market is more than 352,800 as of November, 2011 [152]. Overall, the Apple’s App Store is currently leading in terms of the application store size; however, the growth rate is much slower than Android Market.

The total number of applications in application stores (especially in the Apple’s *App Store* (iOS) and Google’s *Android Market*) is growing very fast (Figure 6) [141-156]. According to the latest “Apple Press Info” on Apples’ App Store, there are more than 425,000 applications as of July, 2011 [141]. The Google’s Android Market has more than 352,800 applications as of November, 2011 according to the recent update from Distimo [152]. The total number of applications for other four OS platforms is very low. As of March 2011, a total of 29,920 applications are available in the Ovi store (Symbian OS), 26,771 in the BlackBerry App World, 11,731 in the MarketPlace (Windows Phone), and 6,363 in the Palm App Catalog [151]. Overall, the iOS leads in the number of applications, but its growth rate is much slower than Android’s (Figure 6). As seen in Figures 3, 4 and 5, Android is currently leading both of the U.S. and Global market share, and the growth rate of its application stores is almost proportional to the increase in market share during the period of 2010 – 2011. Android also leads in the total number of free applications, and has become an increasingly popular competitor of iOS (for iPhone) [151,157]. The market share of iOS is almost consistent during the period of 2010 – 2011 though its application store size increased (Refer to Figures 3, 4 and 5 for details).

## Appendix II: Healthcare Applications for Smartphones

In this study, a total of 83 smartphone-based healthcare applications were discussed. These applications were grouped by the targeted user of the applications, as divided into three groups: (1) 57 applications for healthcare professionals, (2) 11 applications for medical or nursing students, and (3) 15 applications for patients. The functionalities of the applications and supported smartphone platforms were discussed and presented in tabular format. Tables 3, 4, 5, 6, 7, 8, 9 and 10 presents a total of 57 applications for healthcare professionals; Table 3: 21 disease diagnosis applications, Table 4: 6 drug reference applications, Table 5: 8 medical calculator applications, Table 6: 6 literature search applications, Table 7: 3 clinical communication applications, Table 8: 4 HIS client applications, Table 9: 2 medical training applications, and Table 10: 7 general healthcare applications. The applications for medical and nursing students and patients are listed in Tables 11 and 12 respectively.

**Table 3:** Disease Diagnosis Applications

**Application**	**Version (price)**	**Platforms**	**Description**	**Functions**
Johns Hopkins Antibiotic Guide ^URL1^[11,39,46,47]	1.10.4 ($39.95)	iOS, Android, Palm OS, Windows Mobile, BlackBerry	An application to help clinicians in the diagnosis and treatment of infectious diseases.	Includes information on anti-microbial agents, infectious diseases, and commonly- encountered pathogens; provides expert recommendations, evidence-based recommendations, drug lists, and drug-drug interactions.
5MCC^URL2^[43,46]	2011 ($99.95 – 1 year)	Android, BlackBerry, iOS, Palm OS, Windows Mobile	The handheld version of the 5-Minute Clinical Consult (5MCC) guide.	Includes information about diagnosis, treatment, medications, follow-up, and associated conditions for 900-common medical conditions; treatment algorithms such as Acromegaly, Swine Flu (H1N1), etc.; flowcharts of related algorithms and protocols; drug therapy section in each topic; ICD-9-CM code index.
5-Minute Infectious Diseases Consult^URL3^[39,46]	($89.95 – 1 year)	Android, BlackBerry, iOS, Palm OS, Windows Mobile	An application for clinical diagnosis, laboratory testing, and appropriate therapy of infectious diseases.	Includes more than 500 topics; topics are arranged alphabetically.
Sanford Guide to Antimicrobial Therapy ^URL4^[11,16,46,47]	2011 ($29.99)	Palm OS, Windows Mobile, iOS, BlackBerry.	The pocket edition of the Sanford Guide to Antimicrobial Therapy.	Home, back and search buttons; four rapid reference categories; diseases and clinical conditions are organized by site of infection and organism; drug information is organized by type; activity spectra comparison table (color coded) for bacteria, fungi and viruses; internal links for efficient navigation.
ePocrates ID ^URL5^[39,46,47]	Rx Pro ($99.00 – 1 year)	Palm OS, Windows Mobile, iOS, BlackBerry, Android	An infectious-disease application for smartphones; part of ePocrates Rx Pro.	Provides information on more than 300 infections, 350 pathogens and 250 drugs; alphabetical list or search of anatomic location, infection, bug or drug; information on pathogen specific drug therapy, history and diagnosis of infection, drug interactions, adverse reactions, basic pharmacological information, and drug cost; references to review articles; ability to record personal notes.
Infectious Diseases Notes ^URL6^[47]	($39.99)	Palm OS	An application to help clinicians in infectious disease treatment.	Includes organisms and treatments, prophylactic therapy, antimicrobial spectra index, and normal flora index.
UpToDate ^URL7^[11,16,17,40,45]	($495 – 1 Year)	iOS, web-enabled smartphone	An online tool providing evidence-based clinical information (e.g. answering clinical questions, making treatment recommendations)	Includes more than 9,000 physicians topics, about 5,000 drug topics, patient information, “what’s new” alerts, practice-changing updates, abstracts, CME^a^ credit accrual, search filters (adult, pediatric, patient), auto-completion of search terms.
Pocket Medicine Infectious Diseases ^URL8^[46]	3.0 ($50.00)	Palm OS, Windows Mobile	An application that answers questions about disease diagnosis.	Suggestions for therapy; subjects are categorized as history and physical, tests, differential diagnosis, management, specific therapy, follow-up, complications, and prognosis; “what to do first” guidance.
Palm LabDX [44]	Prototype	Palm OS, Windows CE	An application containing information on 193 common laboratory tests.	Displays alphabetical listing of tests; search by test name; display test information such as reference range; causes for increased and decreased values, descriptions of tests, and notes on interpretation and related tests.
Normal Lab Values ^URL9^[26]	1.4 ($1.99)	iOS	An application to help interpret laboratory test results.	Shows reference values both in traditional and SI^b^ units, visualizes labs by categories or alphabetical list, search field.
Lab Unit Converter ^URL10^[26]	1.2 ($4.99)	iOS	An application to convert lab values between conventional and SI^b^ units.	250 common lab tests; quick access to frequently used conversions; search lab tests.
Labs 360^URL11^[26]	($49.95 – 1 year standard subscription)	Android, BlackBerry, iOS, Palm OS, Windows Mobile.	A laboratory guide edited by a practicing clinician.	Includes all common laboratory tests, providing high and low values; cross-reference all skyscape resources; updates every 4–6 weeks.
Davis’s Laboratory and Diagnostic Tests ^URL12^[26]	($49.95)	Android, BlackBerry, iOS, Palm OS, Windows Mobile.	A nursing-focused laboratory and diagnostic test reference.	Provides test procedure information on over 400 labs; patient care before, during, and after the test; RSS feeds of clinical lab-product news; list of drug-test interactions; sub-specialty information.
Pocket Guide to Diagnostic Tests ^URL13^[17,26]	($39.95 – 1 year standard subscription)	Android, BlackBerry, iOS, Palm OS, Windows Mobile.	A diagnostic and laboratory test reference designed for medical, nursing and other health professional students.	Includes laboratory procedures in clinical settings; laboratory tests, diagnostic imaging tests, costs and risks of various procedure; flowcharts of complex algorithms; color images; cross-reference to all other Skyscape applications.
IDdx ^URL14^[23]	1.10 ($1.99)	iOS, Palm OS, Windows Mobile, BlackBerry	A decision support software tool to help medical practitioners to diagnose infectious diseases.	Explores 275 diseases in 15 categories; search by disease name or disease criteria (includes 119 signs and symptoms, 39 epidemiological factors, 16 regions of the world); examples of epidemiological factors; access diseases worldwide; drill down to the infections associated with compromised hosts or bioterrorism.
eRoentgen Radiology Dx ^URL15^[23]	($19.99)	iOS	A smartphone application that helps radiologists to identify the most appropriate radiology exam for a patient.	Identifies most appropriate radiology exam for a patient; informs choice of the best test the first time around; searches by diagnosis and symptoms.
iSeismometer ^URL16^[48]	1.3 (free)	iOS, Windows Phone	A tool for rapid measurement of tremor frequency using the iPhone accelerometer.	Measure and display of movement in X, Y, Z axis and their predominant frequency band.
Video Laser Level ^URL17^[49]	1.0 ($1.99)	iOS	Positions virtual red horizontal lines over the live video.	Allows oculoplastic surgeons to evaluate alignment and misalignment of canthal position during surgical planning, execution, and follow-up.
EyeChart ^URL18^[41]	1.1 (free)	iOS	An application for visual acuity tests.	Includes the Snellen eye chart that is used by eye care professionals to measure visual acuity.
EyePhone ^URL19^[42]	1.0 ($25.00)	iOS	An application for visual acuity test.	Distance E-test, near visual acuity test, fixating, flash light, color test, Amsler grid, and pupil diameter test.
DizzyFIX ^URL20^[41]	1.3 ($14.99)	iOS	Assists clinicians in correctly diagnosing and treating vertigo due to BPPV^c^.	Assists in doing Epley Maneuver, which is recognized as the primary treatment of BPPV^c^, assists clinicians in guiding patient through the correct series of precise head and body positions; video tutorial is available at http://www.youtube.com/v/nDTDRocgFKQ&hl=en_US&fs=1

This table presents the version, platforms, a short description, and a list of functions of the 21 disease diagnosis applications for healthcare professionals.

^a^**CME:** Continuing Medical Education, ^b^**SI**: International System of Units, ^c^**BPPV**: Benign Paroxysmal Positional Vertigo.

Website URLs:

URL1: http://hopkins-abxguide.org, Accessed June, 2011.

URL2: http://www.skyscape.com/estore/ProductDetail.aspx?ProductId=2711, Accessed June, 2011.

URL3: http://www.skyscape.com/estore/productdetail.aspx?productid=265, Accessed June, 2011.

URL4: http://www.sanfordguide.com, Accessed June, 2011.

URL5: http://www.epocrates.com/products/rxpro/index.html, Accessed June, 2011.

URL6: http://pdamedicalsolutions.com/products/idn.htm, Accessed June, 2011.

URL7: http://www.uptodate.com/home/about/mobile-access.html, Accessed June, 2011.

URL8: http://www.pocketmedicine.com/?q=products, Accessed June, 2011.

URL9: http://doctorcalc.com/normal-lab-values, Accessed June, 2011.

URL10: http://doctorcalc.com/lab-unit-converter, Accessed June, 2011.

URL11: http://www.skyscape.com/estore/ProductDetail.aspx?ProductId=2044, Accessed June, 2011.

URL12: http://www.unboundmedicine.com/products/davis_labs_diagnostic_tests, Accessed June, 2011.

URL13: http://www.skyscape.com/estore/ProductDetail.aspx?ProductId=2502, Accessed June, 2011.

URL14: http://www.iddx.info/, Accessed June, 2011.

URL15: http://www.iatrossoftware.com/, Accessed June, 2011.

URL16: http://www.iseismometer.com/category/apps/, Accessed June, 2011.

URL17: http://itunes.apple.com/us/app/video-laser-level/id331550022?mt=8, Accessed June, 2011.

URL18: http://www.dokcompany.com/products, Accessed June, 2011.

URL19: http://www.eyephone.com.br/Eyephone/Home.html, Accessed June, 2011.

URL20: http://www.dizzyfix.com/professionals/dizzyfix-iphone, Accessed June, 2011.

**Table 4:** Drug Reference Applications

**Application**	**Version (price)**	**Platforms**	**Description**	**Functions**
Skyscape’s RxDrugs^URL21^[39]	1.1 (free)	Android, BlackBerry, iOS	An application that provides dosing guidelines for drugs.	Includes thousands of brand-name and generic drugs and dosages; drug-drug interactions with multi-drug analyzer tool; access medications by indication, pharmacologic class or by generic or U.S. or Canadian brand name; integrated weight-based drug dosing calculators.
Epocrates ^URL22^[11,39,40,43,50,51,53]	3.18 (free)	Palm OS, Windows Mobile, iOS, BlackBerry, Android	A drug database application that is part of Epocrates Rx, which is a free product.	Provides clinical information on thousands of prescription medicines; formulary information; identify pills by entering physical characteristics and imprint code; multi-drug interaction checker.
Medscape ^URL23^[39]	2.4.1 (free)	iOS, BlackBerry, Android	A drug reference application for smartphones.	Includes comprehensive drug reference, drug interaction checker, disease and condition reference and treatment guide, procedures reference, daily medical news and alerts, physician, pharmacies, and hospital directories.
SafeMed Pocket ^URL24^[52]	2.0	Windows Mobile	An application that provides access to data on all pharmaceuticals that are sold in Sweden.	Contains drug listings from the FASS (an encyclopedia that is equivalent to American Physician’s Desk Reference, containing detailed information of the medicines that are licensed for sale in Sweden), ICD-10 codes, medical literature, and pharmaceuticals interactions.
FDA Drugs ^URL25^[39]	1.8 ($2.99)	iOS	A tool that provides authoritative info for FDA^c^ drug approvals.	Includes 16,466 approvals for 25,881 drug products since 1939; free monthly updates; search generic drugs for brand name drugs and vice versa; search drug name and active ingredient; covers all drugs from the Orange Book; strength, manufacturer, FDA^c^ approval date, package inserts (description, clinical pharmacology, etc.).
DrugDoses.net ^URL26^[39]	2.0 ($19.99)	iOS, Windows Mobile, Android, Palm OS	A smartphone version of Frank Shann’s booklet on drug dosages for children and adult.	Contains more than 2000 drug dosages for both children and adults; search by drug name; integrates PedCalc (pediatric score and formula calculator) and resuscitation dose calculator for children.

This table presents the version, platforms, a short description, and a list of functions of the six drug reference applications for healthcare professionals.

^d^**FDA**: Food and Drug Administration.

Website URLs:

URL21: http://www.skyscape.com/estore/productdetail.aspx?productid=1093, Accessed June, 2011.

URL22: http://www.epocrates.com/products/rx/, Accessed June, 2011.

URL23: http://www.medscape.com/public/mobileapp, Accessed June, 2011.

URL24: http://www.pharmtech.se/en/broschyrer/SafeMedPocket_Pharmtech_Eng.pdf, Accessed June, 2011.

URL25: http://sigmaphone.com/app.html#FDA, Accessed June, 2011.

URL26: http://www.drugdoses.net/, Accessed June, 2011.

**Table 5:** Medical Calculator Applications

**Application**	**Version (price)**	**Platforms**	**Description**	**Functions**
Epocrates MedMath ^URL27^[11,43,47]	3.18 (free)	Palm OS, Windows Mobile, iOS, BlackBerry, Android	A medical calculator application that is part of Epocrates Rx and available for free.	Provides useful medical formula calculator including pregnancy wheel, and basal energy expenditure etc.
MedCalc ^URL28^[11,42,43,55]	2.3 ($0.99)	iOS	An application that provides medical formula calculator.	More than 200 medical formulas, scores, scales, and classifications, detailed information and bibliographic references for each formula,, support for U.S. and SI^e^ units, search for equations by name or keywords, customizable list of favorite equation.
Medical Calculator ^URL29^[26,41]	1.9 ($0.99)	iOS	An application that compute useful medical formulas and equations.	Includes common formulas and equations, supports U.S. and SI^e^ units.
Calculate ^URL30^[26]	1.3 (free)	iOS, BlackBerry, Android	A medical formula/equation calculator and decision support tool.	Supports SI^e^ and imperial units, detailed references with PubMed integration, navigate calculators by specialty.
Archimedes ^URL31^[16,39]	(free)	Android, BlackBerry, iOS, Palm OS, Windows Mobile	Smartphone version of Archimedes online medical calculator.	This application includes more than 150 commonly used medical formulas, calculator selection through multiple indexes, use conventional (U.S.) or SI^e^ units, and formula details and explanations.
uBurn Lite ^URL32^[56]	3.1.2 (free)	iOS	An application to calculate percent burn of body surface area.	Calculate percent burn of body surface area for adult and children, and parkland formula.
Softforce’s Antobiotic Dosage Calculator ^URL33^[39]	1.2($1.99)	iOS	A drug dosage calculator for the treatment of patients with renal failure.	Calculation based on cockroft-gault formula, dosage required for a particular drug, and dosage adjustment.
Paeds ED ^URL34^[41]	1.0.7 (free)	iOS	A drug dosage calculator to be used by the Pediatrician.	Use guesstimate formula to calculate weight of children from their age in years, calculate correct doses of various drugs based on children’s weight.

This table presents the version, platforms, a short description, and a list of functions of the eight medical calculator applications for healthcare professionals.

^e^**SI**: International System of Units.

Website URLs:

URL27: http://www.epocrates.com/products/rx/, Accessed June, 2011.

URL28: http://medcalc.medserver.be/iphone_description.html, Accessed June, 2011.

URL29: http://doctorcalc.com/medcalc, Accessed June, 2011.

URL30: http://www.qxmd.com/apps/calculate-by-qxmd, Accessed June, 2011.

URL31: http://www.skyscape.com/estore/productdetail.aspx?productid=227, Accessed June, 2011.

URL32: http://www.uburnapps.com/uBurn/uBurn_Lite.html, Accessed June, 2011.

URL33: http://www.softforceapps.com/adc/, Accessed June, 2011.

URL34: http://iedapps.com/medical-apps/ied-paediatric-emergency-drugs/, Accessed June, 2011.

**Table 6:** Literature Search Applications

**Application**	**Version (price)**	**Platforms**	**Description**	**Functions**
PubSearch ^URL35^[51]	1.6 (free)	iOS	An application for medical literature searches from PubMed.	Searches PubMed; display search results; sort by authors, title, journal or year; display article’s abstract; bibliographic entry.
PubMed on Tap ^URL36^[41,42,56]	2.6 ($2.99)	iOS	A medical literature search tool for iPhone.	Searches and displays reference information from PubMed; store references in a searchable personal library; email references from within the application; advanced search specifying search field and using logic operators; links to full text articles; remember recent searches; navigation between references.
MD on Tap ^URL37^[50,55,57-59,63-65]	2.1 (free)	Palm OS, Windows CE, Windows Mobile	An application that retrieves MEDLINE citations through Internet connections.	Searches using 3 search engines: PubMed, Essie, and Google; previous query history; save citations as text file; take notes; cluster search results; related articles; auto spell check; links to full-text article.
askMEDLINE ^URL38^[45,60-62]	(free)	Web-enabled smartphone.	A natural language query tool for PubMed/MEDLINE developed by the NLM^f^.	Searches PubMed by entering natural language query; spell checker; handles query in the form of questions or complex phrases; MeSH speller available in “MeSH Speller + askMEDLINE” program that is an extension of askMEDLINE.
PICO ^URL39^[45,60,62]	(free)	Web-enabled smartphone.	A Patient, Intervention, Comparison and Outcome (PICO) search interface for PubMed/MEDLINE developed by the NLM^f^.	Includes 4 text fields: problem, intervention, compare to, and outcome.
Disease Associations ^URL40^[45]	(free)	Web-enabled smartphone.	A search interface for case reports and review of reported cases in PubMed/MEDLINE developed by NLM^f^.	Includes three text entry fields: (1) two text fields for sign, symptom, disease, condition, or procedure joined with AND operator, (2) one text entry associated with that disease, condition, or procedure.

This table presents the version, platforms, a short description, and a list of functions of the six literature search applications for healthcare professionals.

^f^**NLM:** National Library of Medicine.

Website URLs:

URL35: http://www.deathraypizza.com/deathraypizza/PubSearch_Home.html, Accessed June, 2011.

URL36: http://www.referencesontap.com/, Accessed June, 2011.

URL37: http://mdot.nlm.nih.gov/proj/mdot/mdot.php, Accessed June, 2011.

URL38: http://askmedline.nlm.nih.gov/ask/ask.php?from=tbld, Accessed June, 2011.

URL39: http://pubmedhh.nlm.nih.gov/nlmd/pico/piconew.php, Accessed June, 2011.

URL40: http://askmedline.nlm.nih.gov/ask/da.php?from=tbld, Accessed June, 2011.

**Table 7:** Clinical Communication Applications

**Application**	**Version (price)**	**Platforms**	**Description**	**Functions**
Voalté One ^URL41^[51]	1.3.3 (free, but must purchase and install Voalté server)	iOS, BlackBerry	A communication tool within the hospital to simplify communication among clinicians.	Combines phone calls, text messaging, and prioritized alarm alerts in a single device; PBX^g^ integration over VoIP^h^; log and retrieve all alarms.
Amcom Mobile Connect ^URL42^[51]	2.2 (free, but requires a connection to supported Amcom Web/Console call center system)	iOS, Android, BlackBerry	A messaging and alerting application for use in healthcare.	Separates critical email and SMS^i^ messages from less important ones; audit trail that logs time and date along with all messages; acknowledgement of messages.
mVisum ^URL43^[20]	(free, but must purchase and install mVisum Medical Communication System)	iPhone, Android, Windows Mobile, Blackberry	A cardiology communication application for cardiologist’s that receive patient data on smartphone.	Receives monitor data, alarms, ECGs^j^, lab results, echocardiograms*, MRIs^k,^*, discharge notes and other reports. [* These have not yet received FDA^l^ clearance as of June 2011.]

This table presents the version, platforms, a short description, and a list of functions of the three clinical communication applications for healthcare professionals.

^g^**PBX**: Private Branch Exchange, ^h^**VoIP:** Voice over Internet Protocol, ^i^**SMS**: Short Message Service, ^j^**ECG:** Electrocardiography, ^k^**MRI:** Magnetic resonance imaging, ^l^**FDA:** U.S. Food and Drug Administration.

Website URLs:

URL41: http://www.voalte.com/Products.aspx, Accessed June, 2011.

URL42: http://www.amcomsoftware.com/Solutions/smartphone_and_tablet_messaging/, Accessed June, 2011.

URL43: http://www.mvisum.com/mVisumCCS.php, Accessed June, 2011.

**Table 8:** HIS Client Applications

**Application**	**Version (price)**	**Platforms**	**Description**	**Functions**
OsiriX Mobile ^URL44^[22,23,51,68]	2.0.2 ($29.99)	iOS	A DICOM^m^ viewing program that is the client application for OsiriX PACS^n^.	Standard DICOM^m^ query and retrieval; views and processes DICOM^m^ images; zooming, panning, rotation, windowing, and leveling; calibrated distance measurement; Oval ROI^o^ measurements of area and density/signal intensity; image transfer.
MEDITECH ^URL45^[20]	N/A	Web-enabled smartphone.	Access patient record from MEDITECH EMR^p^ system securely on smartphone using a Web browser	Accesses clinical data including lab results, vital signs, intake and output, allergies, active medications, and documents (reports and notes).
PatientKeeper Mobile Clinical Results ^URL46^[20]	N/A	Web-enabled smartphone.	Provides access to patients’ clinical data from PatientKeeper EMR^p^ using a Web browser	Accesses patient list, patient summary, lab results, test results, medication list, clinical notes, problem list, vital signs, allergies, order status etc.; customizes workflow based on each physician’s requirements.
AirStrip OB ^URL47^[23]	1.6(free, but needs to purchase and install Airstrip OB system)	iOS, Android	Provides access of hospital’s Labor and Delivery unit in the EMR to the Obstetricians from smartphone.	Displays real-time and historical waveforms, HIPAA^q^ complaint authentication login, displays the fetal strip and maternal contraction pattern information for an individual patient, access patient data, and zooming and scrolling chart images.

This table presents the version, platforms, a short description, and a list of functions of the four HIS client applications for healthcare professionals.

^m^**DICOM**: Digital Imaging and Communications in Medicine, ^n^**PACS**: Picture archiving and communication system, ^o^**ROI**: Region of Interest, ^p^**EMR**: Electronic Medical Record, ^q^**HIPAA**: Health Insurance Portability and Accountability Act.

**Website URLs:** URL44: http://www.osirix-viewer.com/, Accessed June, 2011.

URL45: http://www.meditech.com/productbriefs/pages/ProductBriefsCSPCM.htm, Accessed June, 2011.

URL46: http://www.patientkeeper.com/products/clinical_applications/clinical_results_mobile.html, Accessed June, 2011.

URL47: http://www.airstriptech.com/Portals/_default/Skins/AirstripSkin/tabid/61/Default.aspx, Accessed June, 2011.

**Table 9:** Medical Training Applications

**Application**	**Version (price)**	**Platforms**	**Description**	**Functions**
iCPR ^URL48^ (D-Sign.it, Bologna, Italy) [71]	Lite-1.3 (free), Full-1.1 (free)	iOS	An application for CPR^r^ training, based on both American Heart Associationand European Resuscitation Council guidelines.	CPR^r^ tutorial; measures chest compression rate; gives audiovisual feedback.
iResus ^URL49^[70]	1.41(free)	iOS	Provides access to UK’s resuscitation guidelines algorithms.	Includes adult and pediatric algorithms; displays algorithms in an intuitive and interactive format; pulls latest algorithms from a central server.

This table presents the version, platforms, a short description, and a list of functions of the two medical training applications for healthcare professionals.

^r^**CPR**: Cardiopulmonary Resuscitation.

**Website URLs:** URL48: http://www.icpr.it/, Accessed June, 2011.

URL49: http://www.imobilemedic.com/productDescription.php?prodID=2, Accessed June, 2011.

**Table 10:** General Healthcare Applications

**Application**	**Version (price)**	**Platforms**	**Description**	**Functions**
HCSIT ^URL50^[72]	2.0 (free)	Palm OS, Windows Mobile	An application to assist clinicians with smoking cessation counseling of patients at the point of care.	Includes Public Health Service guidelines on smoking cessation; smoking cessation drug prescribing information; FTND^s^ questionnaire for scoring nicotine dependence; recommend pharmacotherapy for highly dependent smokers.
Borboleta ^URL51^[73]	1.0.2 (free)	Palm OS, Symbian OS, Windows Mobile, Android	A mobile telehealth system for primary homecare.	Registration of patient home visits; presents patient data, patient caregiver data, patient socioeconomic data, scheduled visits, new visit registration, visit history of the patient, disease catalog, and medication catalog.
LIFe-reader [52]	Not Found	Windows Mobile	A smartphone-based CDSS^t^ application with a barcode reader designed for nurses in geriatric homecare.	Scans EAN^u^ codes on drug packages; obtains patient’s medication profile; checks for inappropriate drugs, drug-drug interactions, therapeutic duplication, and warnings for unsuitable drugs for elderly people.
Multimedia Paging Based Clinical Alarm [75]	Prototype	Web-enabled smartphone	A real-time clinical alarm system to monitor intensive care patients.	Color-based severity indicator; displays the alarms for an intensive care unit; displays vital signs at the moment of alarm; displays all alarms for a patient bed.
Outbreaks Near Me ^URL52^[39]	1.1 (free)	iOS (iPhone, iPod Touch, iPad), Android	An application for real-time disease outbreak information.	Displays outbreak location in Google map and outbreak details from HealthMap database, which utilizes medical email list services, news, media report, official alerts, etc.
H1N1 Swine Flu Update ^URL53^[39]	No longer supported	iOS	A newsreader application for Swine Flu (H1N1) outbreaks.	News feed on H1N1 outbreak from CDC^v^, WHO^w^ and major news organizations.
WISER ^URL54^[74]	(free)	Windows Mobile, Plam OS, iOS, BlackBerry, Android	An application for Emergency Medical Service Specialists that identifies chemical and biological hazards on the basis of symptoms and signs.	Provides access to NLM’s Hazardous Substances Data Bank (HSDB); radiological and biological substance report.

This table presents the version, platforms, a short description, and a list of functions of the seven general healthcare applications. These seven applications were categorized as “general” since they do not fit into any of the categories from Tables 3 4, 5, 6, 7, 8 and 9.

^**s**^**FTND**: Fagerstrom Test for Nicotine Dependence, ^t^**CDSS**: Clinical Decision Support System, ^u^**EAN**: European Article Number, ^v^**CDC:** Center for Disease Control, ^w^**WHO**: World Health Organization .

Website URLs:

URL50: http://www.smokefree.gov/software/Smartphone_Manual_Final.pdf, Accessed June, 2011.

URL51: http://ccsl.ime.usp.br/borboleta/, Accessed June, 2011.

URL52: http://healthmap.org/outbreaksnearme/, Accessed June, 2011.

URL53: http://www.qxmd.com/apps/h1n1-update, Accessed June, 2011.

URL54: http://wiser.nlm.nih.gov/, Accessed November, 2011.

**Table 11:** Medical Education Applications

**Application**	**Version (price)**	**Platforms**	**Description**	**Functions**
I-Surgery Notebook ^URL55^[51]	3.0 ($1.99)	iOS, Android	An application to keep a log of surgical cases and procedures.	Stores data on surgeries including procedure, pre-operative diagnosis, post-operative diagnosis, name of surgeons involved, and type of anesthesia used; search case log.
Eponyms ^URL56^[51]	1.3.1 ($1.99, free student edition)	iOS, Android	Provides details of eponymous signs and diseases.	Includes eponym database from http://www.eponyms.net/; browse eponyms.
Netter’s Atlas of Human Anatomy ^URL57^[23,56]	($76.95 – 1 year)	Android, BlackBerry, iOS, Palm OS, Windows Mobile	A smartphone-based human anatomy tool for medical education purposes.	Contains more than 532 colored anatomic illustration, radiographs, computed tomographic (CT) images, CT angiograms, and MRIs; three dimensional images of human body; separate groups of anatomic illustration, i.e. head and neck, back and spinal cord, thorax, abdomen, pelvis and perineum, upper and lower limb.
Netter’s Anatomy Flash Cards ^URL58^[51,56]	($36.95 – 1 year)	Android, BlackBerry, iOS, Palm OS, Windows Mobile	A mobile application containing about 300 interactive flash cards.	Images with hot-spot help identifying parts of anatomy easily; clinical notes, separate groups of anatomic illustration, i.e. head and neck, back and spinal cord, thorax, abdomen, pelvis and perineum, upper and lower limb.
Blausen Ear Atlas ^URL59^[41]	2.11 ($2.99)	iOS, Android, BlackBerry	An application that provides a set of ear- related video animations.	Includes 6 video animations: cochlear implants, ear pressure, ear tubes, hearing loss, hearing test, otitis media.
Oxford Handbook of Clinical Specialties ^URL60^[41]	1.5 (£34.99 – UK only)	iOS, BlackBerry	Handheld version of the latest edition of Oxford Handbook of Clinical Specialties.	Includes twelve books, fully cross-referenced to the Oxford Handbook of Clinical Medicine, practical advice, cross-checked by authoritative subject experts, color illustrations and clinical photographs.
Dissection ^URL61^[41]	1.3 ($4.99)	iOS	A human anatomy application that mainly focuses on head and neck.	This application displays human dissections; audio enabled annotations; tap-on identification feature; includes head, neck, and thorax.
Cranial Nerves ^URL62^[41]	1.7 ($4.99)	iOS	A learning tool on cranial nerves and skull base.	This application includes the cranial nerves and skull base from high resolution CT scans; interactive visualization; control the position and transparency of the skull and each nerve along with the appropriate textual information.
iSilo ^URL63^[17,47]	5.26 ($19.99)	Palm OS, Windows Mobile, Symbian OS, BlackBerry, iOS, Android	A document reader application on smartphone.	Stores text in a highly compressed format; password protected; categorization of documents; search text within a document or set of documents; copy and paste; navigation includes scrolling, jumping, and marks; maximizing screen by hiding scrollbar, title bar, toolbar, etc.; left-hand support; remember last view location; remember jump history for backtracking; local bookmarks.
Mobipocket Reader ^URL64^[11]	(free)	BlackBerry, Windows Mobile, Symbian OS, Palm OS	An electronic book reader on smartphone.	Customizable display; library view of all eBooks stored in local media; annotate, highlight, bookmark any part of the eBook; search and lookup any word in the dictionary.
Instant ECG ^URL65^[23]	2.7 ($0.99)	iOS	A basic ECG^j^ tutorial application.	Includes ECG^j^ electrophysiology, myocardial action potential, associated waveforms, and intervals and segments.

This table presents the version, platforms, a short description, and a list of functions of the eleven medical education applications for medical and nursing students.

^j^**ECG:** Electrocardiography.

Website URLs:

URL55: http://www.lifewareapps.com/index.php?option=com_content&view=article&id=49&Itemid=2, Accessed June, 2011.

URL56: http://code.google.com/p/eponyms-touch/, Accessed June, 2011.

URL57: http://www.skyscape.com/estore/ProductDetail_Netter5.aspx, Accessed June, 2011.

URL58: http://www.skyscape.com/estore/productdetail.aspx?ProductId=2833, Accessed June, 2011.

URL59: http://blausen.com/products, Accessed June, 2011.

URL60: http://www.medhand.com, Accessed June, 2011.

URL61: http://www.ehuman.com/products/bassett-dissection-iphone, Accessed June, 2011.

URL62: http://www.ehuman.com/products/cranial-nerves-iphone, Accessed June, 2011.

URL63: http://www.isilo.com/info/features.htm, Accessed June, 2011.

URL64: http://www.mobipocket.com/en/downloadsoft/productdetailsreader.asp, Accessed June, 2011.

URL65: http://www.instantecg.org/instant-ecg/, Accessed June, 2011.

**Table 12:** Applications for Patients

**Application**	**Version (price)**	**Platforms**	**Description**	**Functions**
Diabeo ^URL66^[32]	Beta	Windows Mobile	A telemedicine solution for diabetes management.	Includes bolus calculators using validated algorithms; takes into account carbohydrate intake, pre-meal blood glucose, and anticipated physical activity reported; plasma glucose targets; automatic adjustments of carbohydrate ratio and basal insulin; data transmission to medical staff computers through GPRS^x^.
Cardiomobile ^URL67^[35]	Prototype	Windows Mobile	A real-time remote monitoring system for cardiac rehabilitation.	Sends ECG^j^ rate, walking speed, heart rate, elapsed distance and patient location to a secure server via GPRS^x^ during exercise sessions; server-side software displays these data.
Pulmonary Rehabilitation [29]	Prototype	Windows Mobile	An application based on standard pulmonary rehabilitation program for self-management, consists of Bluetooth pulse oximeter and smartphone.	Select and start exercise program; set custom personalized upper and lower heart rate; display heart rate, time remaining in seconds and feedback color (green: normal physical condition, amber: normal condition but near acceptable limits, red: dangerous physical condition).
PAL Calculator [82]	Prototype	Java-enabled smartphones	Measures physical activity level (PAL) through questionnaire application on smartphones.	Displays questionnaire; measures PAL.
Asthma Peak Flow Monitoring [79]	Prototype	Windows Mobile	An application to monitor peak flow of asthma patients.	Sends peak-flow reading through GPRS^x^ network to a secure server; receives asthma trend analysis feedback from the server.
eCAALYX [83]	Prototype	Android	A remote monitoring system for older people with multiple chronic conditions.	Receives data from wearable health sensors in a smart garment; transmits data to the monitoring server; identifies higher-level information such as tachycardia and signs of respiratory infections based on established medical knowledge; displays most recent medical details obtained from the sensors.
Hearing Check ^URL68^[86]	1.0 (free)	iOS	A simple and confidential hearing check tool developed by RNID^y^, UK	Calls a landline number to access a free hearing check.
uHear ^URL69^[41]	1.0 (Free)	iOS	A hearing loss self-assessment test.	Three assessments: hearing sensitivity, speech in noise, and a questionnaire about common listening situations.
iTinnitus ^URL70^[41]	1.51 ($4.99)	iOS	A sound therapy package for patients with tinnitus.	Records tinnitus by frequency in Hertz and keeps track of the tinnitus in a daily diary graph, supports full masking therapy that is some form of sound played at a volume around the same volume as the patient’s tinnitus, also supports residual inhibition and habituation.
Sleep Aid ^URL71^[41]	1.3 ($2.99)	iOS	A sleep apnea management application.	Records snoring; generates graph of snoring; plays back snoring sound; provides information about sleep apnea.
Fall Detector [78]	Prototype	Smartphone with camera support.	A fall detection system consisting of tri-axial accelerometer with embedded processor and camera phone.	The embedded processor in the accelerometer process the data locally, sends data to the camera phone through a Bluetooth connection during a suspected fall; phone generates a request to the user for vocal or keypad response; automatically calls emergency services in serious situations; sends data and video to emergency services through high-speed 3G network.
Fall Detector [80]	Prototype	Wireless-enabled smartphone.	A fall detection application consisting of a smartphone with wireless Internet connection, tri-axial accelerometer, and microcontroller.	Detects a fall; sends data to the server for further analysis to determine an emergency.
iFall [81]	Prototype	Android	An application for fall detection and response.	Detects falls; determines false positives, request user’s attention by vibrating the phone, flashing LEDs and screen, playing an audio message; makes automatic emergency-services call.
Purdue Momentary Assessment Tool ^URL72^[84,85,87]	2.1.3 (free)	Palm OS	A human behavior monitoring tool.	Event-driven study design; the application generate a beep when an event is fired; displays question sets, sets do-not-disturb time during busy moments.
Mayo Clinic Meditation ^URL73^[23]	1.0 ($2.99)	iOS	An application that helps to practice meditation.	Includes short training videos introducing key concepts; 15-minute meditation program and 5-minute meditation program.

This table presents the version, platforms, a short description, and a list of functions of the 15 applications for patients. Of these, six applications are for disease management with chronic condition, four are ENT-related, three are fall-related, and two other conditions.

^j^**ECG:** Electrocardiography, ^x^**GPRS**: General Packet Radio Service, ^y^**RNID**: Royal National Institute for Deaf People.

Website URLs:

URL66: http://www.diabeo.com/, Accessed June, 2011.

URL67: https://www.ihbi.qut.edu.au/about/researchover/injury/cardiomobile.jsp, Accessed June, 2011.

URL68: http://www.actiononhearingloss.org.uk/your-hearing/technology/equipment-and-research/iphone-hearing-check.aspx, Accessed June, 2011.

URL69: http://www.unitronhearing.com/unitron/global/en/professional/your_practice/uhear.html, Accessed June, 2011.

URL70: http://www.innerearsolutions.com/Software_Solutions.html, Accessed June, 2011.

URL71: http://www.remoteanalysis.net/sleepaid.html, Accessed June, 2011.

URL72: http://www.ruf.rice.edu/~dbeal/PMAT.html, Accessed June, 2011.

URL73: http://www.mremedy.com/Home_Products.php, Accessed June, 2011.

## Competing interests

The authors declare that they have no competing interests.

## Authors' contributions

ASMM formulated the study design, performed literature search, screened and reviewed the articles satisfying the eligibility criteria, collected data from each eligible article and drafted the manuscript. IY participated in the study design and helped draft the manuscript. LS helped draft the manuscript. All authors read and approved the final manuscript.

## Pre-publication history

The pre-publication history for this paper can be accessed here:

http://www.biomedcentral.com/1472-6947/12/67/prepub
